# Dithiocarbamate-modified cellulose-based sorbents with high storage stability for selective removal of arsenite and hazardous heavy metals†

**DOI:** 10.1039/d0ra05573e

**Published:** 2020-08-17

**Authors:** Futo Morita, Keisuke Nakakubo, Koki Yunoshita, Masaru Endo, Foni B. Biswas, Tatsuya Nishimura, Asami S. Mashio, Hiroshi Hasegawa, Tsuyoshi Taniguchi, Katsuhiro Maeda

**Affiliations:** Graduate School of Natural Science and Technology, Kanazawa University Kakuma-machi Kanazawa 920-1192 Japan hhiroshi@se.kanazawa-u.ac.jp tsuyoshi@p.kanazawa-u.ac.jp maeda@se.kanazawa-u.ac.jp; Daicel Corporation 1239 Shinzaike, Aboshi-ku Himeji Hyogo 671-1283 Japan; Department of Chemistry, University of Chittagong Chittagong 4331 Bangladesh; Nano Life Science Institute (WPI-NanoLSI), Kanazawa University Kakuma-machi Kanazawa 920-1192 Japan

## Abstract

A series of cellulose derivatives bearing dialkyl dithiocarbamate (DTC) groups were synthesized. Their ability of sorption of arsenite (As(iii)) and heavy metals and their storage stability in the solid state were investigated. Among them, DTC-modified cellulose derived from l-proline showed the highest sorption capacity for As(iii) and heavy metals to selectively remove them from aqueous media. It also showed exellent storage stability in air at 40 °C.

## Abstract 2

Dithiocarbamate-modified cellulose derived from l-proline works as a storable sorbent for selective removal of toxic As(iii) and heavy metals from aqueous media.

## Graphical abstract

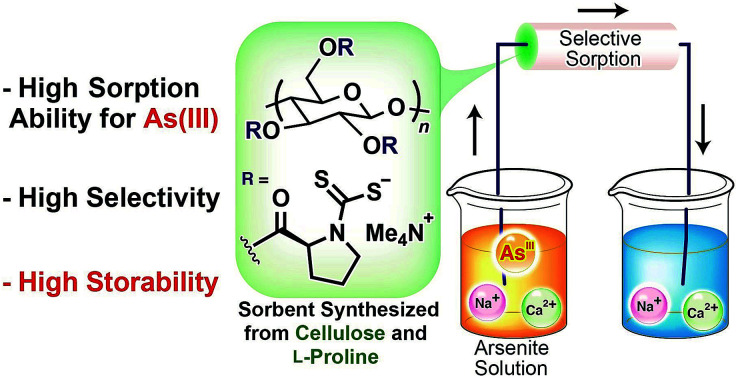

## Introduction

Compounds having a dithiocarbamate (DTC) group work as good chelating agents to capture heavy metals because the DTC group is a soft Lewis base that has strong affinity toward soft Lewis acids such as heavy metals to form stable complexes according to the HSAB rule.^1,2^ Several small organic molecules having a DTC group, such as sodium diethyldithiocarbamate, have been industrially used as sorbents for the removal of hazardous heavy metals from aqueous or organic media.^3,4^ Such small molecule-based sorbents are readily available, but further treatment is often required for the efficient removal of the resultant complexes from aqueous media due to the difficulty in precipitation of the heavy metal complexes.^5^ Polymer-based sorbents carrying a DTC group are potential materials for the removal of heavy metals from aqueous media because they can work as heterogeneous sorbents for solid–liquid extraction and can be easily recovered from water.^6^ However, typical polymer-based sorbents are synthesized from petroleum-based chemical materials, and the production of a large amount of waste acid solutions poses serious problem for the environment.^7^

**Fig. 1:**
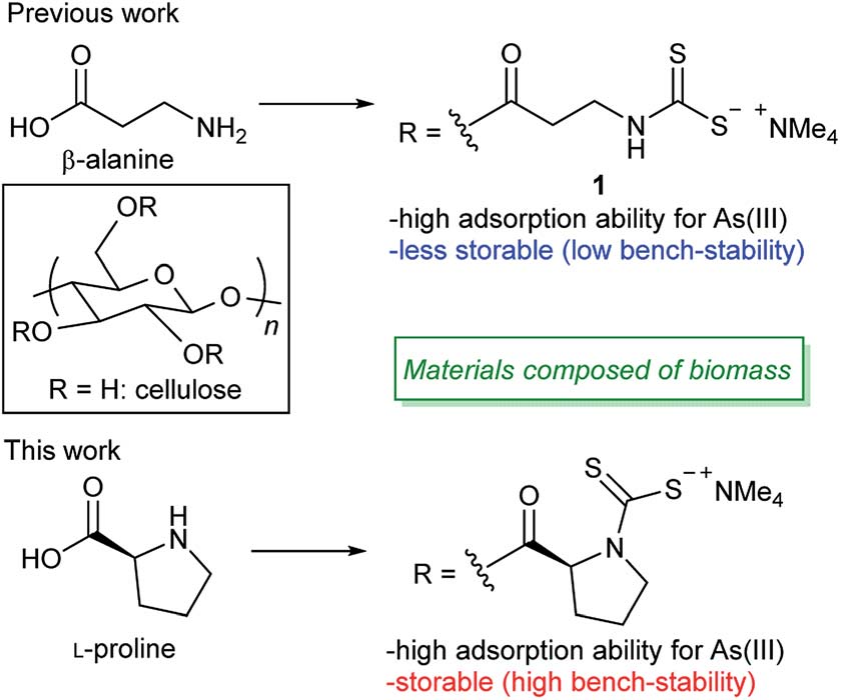
DTC-modified cellulose-based sorbents for the selective removal of As(iii).

Cellulose is the world's most abundant natural polymeric raw material with a fascinating structure and properties.^8^ This polysaccharide is capable of being chemically modified through the hydroxyl groups in order to develop eco-friendly and cost-effective biosorbents for wastewater treatment. Recently, our group synthesized a DTC-modified cellulose material 1 with excellent ability as a selective sorbent for highly toxic arsenite, which is an inorganic As(iii) compound, from aqueous media (Fig. 1).^9,10^

It is generally believed that DTC compounds are stable in the solid state. During the course of the above study, however, we found that the sorption capacity of compound 1 for As(iii) significantly decreased with time when it was stored even in the solid state under ambient conditions, probably due to decomposition of the DTC groups by moisture or oxygen. Although degradation behaviors of polymer-based sorbents having DTC groups in the solid state have not been investigated in detail, degradation might be a general problem for DTC-modified polymer-based sorbents. This drawback would greatly limit the applicability of the material because manufacturing and transport of materials with poor storage stability are problematic.

We therefore decided to develop DTC-modified cellulose materials with good storage stability that are capable of efficiently removing toxic As(iii) and heavy metals from aqueous media. Since monoalkyl DTC compounds (R–NH–C(=S)S^−^; R = alkyl group) have several competitive decomposition pathways based on the N–H group,^11^ they do not have sufficient stability compared with the stability of dialkyl DTC compounds. In this study, we synthesized a series of dialkyl DTC-modified (–R^1^–N (R^2^)–C(=S)S^−^; R^1^ and R^2^ = alkyl group) cellulose materials and evaluated their ability for sorption of As(iii) and other heavy metals as well as their storage stability. As a result, we identified a novel biopolymer material derived from cellulose and l-proline as a potential sorbent with excellent storage stability for selective sorption of As(iii) and other heavy metals (Fig. 1).

## Results and discussion

**Fig. 2:**
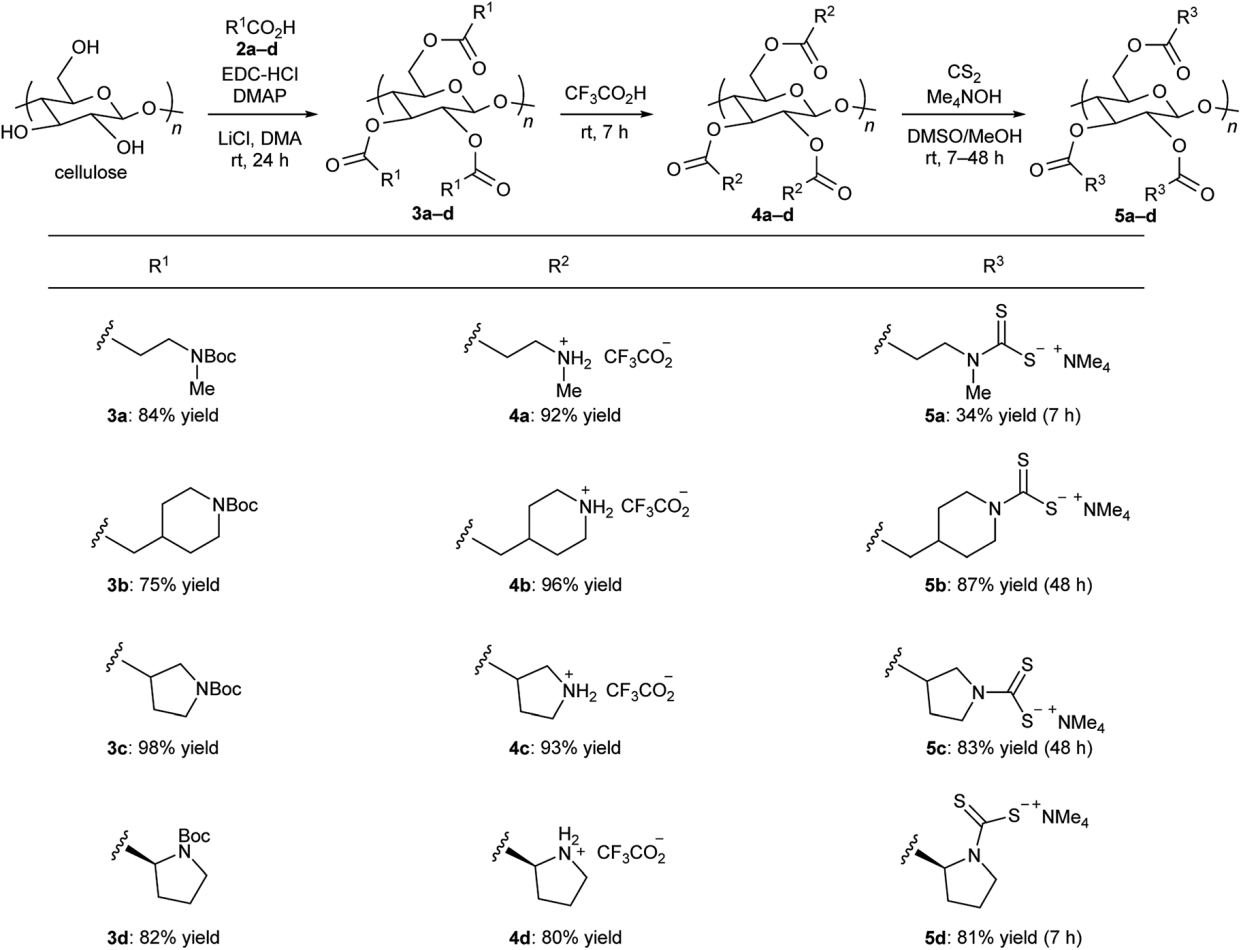
Synthesis of DTC-modified cellulose derivatives 5a–d.

We began our study with the design and synthesis of four new DTC-modified cellulose compounds 5a–d (Fig. 2). First, commercially available microcrystalline cellulose was converted to the corresponding cellulose esters 3a–d by condensation between acyclic and cyclic N-protected amino acid derivatives 2a–d in the presence of 1-ethyl-3-(3-dimethylaminopropyl)carbodiimide hydrochloride (EDC-HCl) and 4-(*N*,*N*-dimethylamino)pyridine (DMAP). Compounds 3a–d were readily soluble in organic solvents, and ^1^H NMR and elemental analyses of 3a–d indicated that the degree of substitution (DS) was almost 3. Next, the *tert*-butoxycarbonyl group of 3a–d was removed by treatment with trifluoroacetic acid (TFA) to give secondary ammonium salts 4a–d. Finally, treatment of 4a–d with CS_2_ and tetramethylammonium hydroxide provided the corresponding dialkyl DTC-modified cellulose compounds 5a–d as white powders in good yields.§§An appropriate caution (*e.g.*, the use of safety glass and glove) should be paid for the use of potentially toxic reagents such as DMAP and Me_4_NOH. In addition, the use of hazardous CS_2_ as a reagent is unavoidable for the synthesis of DTC compounds, but reacted CS_2_ is essentially incorporated to the material as a stable DTC group. Since compounds 5a–d were slightly soluble in water or acetic acid, ^1^H NMR analysis of these compounds could be performed unlike in our previous study.^9^ Although ^1^H NMR spectra of 5a–d showed rather broadened signals, they clearly indicated the existence of each side chain and tetramethylammonium cation (see ESI†). IR spectra of 5a–d showed a typical and strong band at around 1730–1740 cm^−1^ based on C=O stretching of ester groups. The spectra also displayed characteristic bands at around 1375–1485 cm^−1^ and 1155–1185 cm^−1^, which correspond to N–C(S) and C=S stretching of DTC groups, respectively. The N–C(S) vibration characteristically shifted to a lower value in the order of acyclic 5a (1485 cm^−1^) > 6-membered cyclic 5b (1410 cm^−1^) > 5-membered cyclic 5c (1384 cm^−1^) and 5d (1375 cm^−1^) (see ESI†).

Next, we evaluated the sorption ability of the obtained dialkyl DTC-modified cellulose compounds 5a–d for As(iii) (Table 1). Our previous study showed that the monoalkyl DTC-modified cellulose 1 had high capacity for sorption of As(iii) in acidic and neutral conditions (≤pH 7) because As(iii) exists as a neutral form over a wide pH range (p*K*_a1_ = 9.2),^9^ and As-containing wastewater such as mining and smelting wastewater is usually acidic.^12^ Therefore, we tentatively compared the sorption capacities of compounds 5a–d for As(iii) at pH 3. Compound 5a, which is an N-methylated analogue of 1, showed a lower sorption capacity than 1 (Table 1, entries 1 and 2). The sorption capacity of compound 5b having a piperidine ring was also not good (Table 1, entry 3). Compound 5c having a pyrrolidine ring showed better sorption capacity than 5a or 5b, but its sorption capacity was still lower than that of 1 (Table 1, entry 4). Finally, we found that the sorption capacity of compound 5d having a l-proline side chain is superior to that of 1 (Table 1, entry 5). The impact of reaction time in the introduction of DTC groups to 4d does not seem to be significant because 5d synthesized from 4d in a prolonged reaction time (7 h → 24 h) did not show improved sorption capacity for As(iii) (503.6 ± 65.8 μmol g^−1^).

**Table 1:** Sorption capacity of 1 and 5a–d for As(iii)

Entry	Sorbent	Amounts of adsorbed As(iii) (μmol g^−1^)^a^	
		As is	After 2 weeks^b^
1	1	595.3 ± 5.1	66.5 ± 6.2 (−89%)
2	5a	480.6 ± 20.9	430.2 ± 7.6 (−11%)
3	5b	332.1 ± 2.6	184.0 ± 2.4 (−45%)
4	5c	489.5 ± 40.9	464.8 ± 1.5 (−5%)
5	5d	618.8 ± 17.6	595.2 ± 11.7 (−4%)

a Conditions: [As(iii)] = 2 mmol L^−1^ at pH 3.

b Under air at 40 °C. Percentage values in parentheses show a rate of decrease in the sorption capacity.

We investigated the change in sorption capacity of compounds 1 and 5a–d after they had been stored in air at 40 °C for 2 weeks (Table 1). The sorption capacity of compound 1 having an N–H group was significantly decreased by about 89% after 2 weeks (Table 1, entry 1), and this was consistent with the gradual degradation observed in the course of storage under ambient conditions as mentioned in the introduction section. On the other hand, the sorption capacity of N-substituted DTC derivatives 5a–d did not decrease as much as that of compound 1 even after 2 weeks (Table 1, entries 2–5). Notably, the sorption capacity of 5c and 5d having a pyrrolidine moiety was hardly changed after 2 weeks (Table 1, entries 4 and 5), indicating that these sorbents are sufficiently stable to be stored for a long time under ambient conditions.¶¶After exposing the materials to air at 40 °C for 2 weeks, the IR spectrum of 1 greatly changed whereas those of 5d hardly changed (Fig. S1 and S2†). Consequently, 5d having a l-proline side chain was identified as a practical DTC-modified cellulose-based sorbent with both high sorption capacity and excellent storage stability. These good properties of 5d might be due to the high nucleophilicity of a pyrrolidine moiety, which could strongly ligate CS_2_.^13,14^ However, it is unclear why the sorption capacity of 5d is different.

**Fig. 3:**
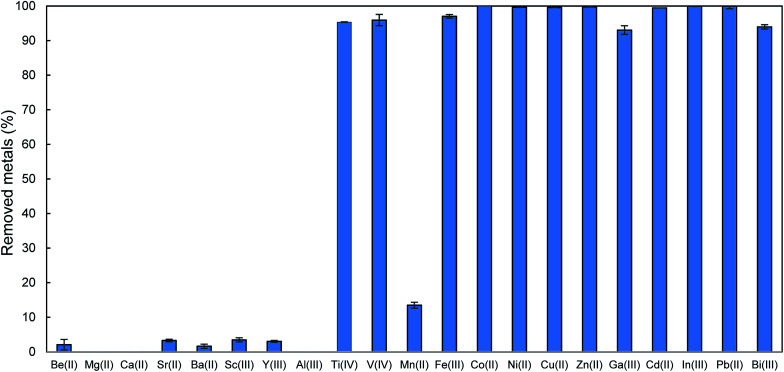
Removal percentages of metals from 5 mg L^−1^ multi metal solution at pH 3 using 5d.

Finally, we preliminary tested the efficiency of compound 5d for the removal of other metal ions using aqueous solutions containing 21 representative metal ions together with As(iii) as shown in Fig. 3. Compound 5d adsorbed heavy metals, including V(iv), Fe(iii), Co(ii), Ni(ii), Cu(ii), Zn(ii), Ga(iii), Cd(ii), In(iii), Pb(ii) and Bi(iii), with high efficiency.^1,9^ In contrast, compound 5d hardly adsorbed alkaline earth metal ions, which are hard metals. This trend is very similar to that of 1 and is consistent with the HSAB rule.^2^ Exceptionally, Ti(iv), which is a hard acid, was efficiently adsorbed by 5d, and this might be because Ti(iv) could form a stable multidentate complex with DTC groups like other heavy metals.^15^ Thus, 5d is a potential sorbent for the selective and efficient removal of As(iii) and other hazardous heavy metals from natural water or wastewater with high concentrations of hard metal ions such as alkaline earth metal ions.

## Conclusions

We have developed a new dialkyl DTC-modified biomass-based sorbent 5d derived from cellulose and l-proline. This sorbent is a potential material for the selective removal of toxic As(iii) and other heavy metals from aqueous media because it has high capacity for As(iii) and other hazardous heavy metals but hardly adsorbs alkaline earth metal ions. The sorption capacity and stability was maintained even after exposure to air at 40 °C for 2 weeks, indicating its excellent storage stability for practical use. In contrast, a significant decrease in the sorption capacity for As(iii) was observed for monoalkyl DTC-modified compound 1, suggesting that monoalkyl DTC-modified polymer-based sorbents might have poor storage ability, and caution is therefore required for the practical use of such materials. The present study demonstrated development of an improved sorbent for the selective removal of As(iii) and other heavy metals based on a solid molecular design with biopolymer. Studies for further improvement and practical applications of the material are ongoing in our laboratory.

## Experimental

### 1. General remarks

#### 1.1. For synthetic experiments

All reactions were performed in oven-dried glassware. All reagents purchased commercially were used without further purification unless otherwise noted. Dehydrated solvents were purchased from Kanto Chemical Co., Inc. Cellulose (Avicel, DP: *ca.* 200) was purchased from Merck. *N*-(*tert*-Butoxycarbonyl)-*N*-Me-β-alanine (2a) was prepared by methylation of commercially available *N*-(*tert*-butoxycarbonyl)-β-alanine with iodomethane.^16^ 2-(1-(*tert*-Butoxycarbonyl)piperidin-4-yl)acetic acid (2b), 1-(*tert*-butoxycarbonyl)pyrrolidine-3-carboxylic acid (2c), (*tert*-butoxycarbonyl)-l-proline (2d) and tetramethylammonium hydroxide (Me_4_NOH, 10% in methanol) were purchased from Tokyo Chemical Industry Co., Ltd. (TCI). Lithium chloride, 1-ethyl-3-(3-dimethylaminopropyl)carbodiimide hydrochloride (EDC-HCl), *N*,*N*-dimethyl-4-aminopyridine (DMAP), trifluoroacetic acid (TFA), and carbon disulfide were purchased from Wako Pure Chemical. DTC-modified cellulose 1 was prepared according to the procedure previously reported.^9^

^1^H NMR spectra were recorded on Bruker Avance 400 and JEOL JNM-ECA 500 spectrometers at 20 °C unless otherwise noted. Chemical shifts (*δ*) are quoted relative to tetramethylsilane (^1^H NMR, *δ* 0 ppm) or a solvent residual peak (D_2_O: *δ* 4.79 ppm; CD_3_CO_2_D: *δ* 2.04 ppm) as the internal standard. Coupling constants (*J*) are given in Hz. Multiplicities are indicated as follows: s (singlet), d (doublet), t (triplet), q (quartet), m (multiplet), or br (broadened). IR spectra were recorded with a JASCO Fourier Transform IR-4700 spectrophotometer. Elemental analyses were performed by the Research Institute for Instrumental Analysis of Advanced Science Research Center, Kanazawa University or by the Research Initiative Center, Tottori University.

#### 1.2. For batch sorption experiments

All laboratory wares were soaked in an alkaline detergent (Scat 20X-PF; Nacalai Tesque) overnight, and then rinsed with deionized water. Subsequently, they were soaked in 3 mol L^−1^ HCl overnight, and then washed again with deionized water. As(iii) standard solution (1000 mg L^−1^), sodium hydroxide (NaOH), nitric acid (HNO_3_, 60%) and acetic acid (AcOH, 99%) were purchased from Kanto Chemical. Sodium acetate (AcONa) was purchased from Nacalai Tesque. ICP multi-element standard solution IV containing 21 elements (Al, Ba, Be, Bi, Ca, Cd, Co, Cu, Fe, Ga, In, K, Li, Mg, Mn, Na, Ni, Pb, Sr, Y, Zn) was purchased from GL science.

The metal concentrations were quantified with inductively coupled plasma optical emission spectrometry (ICP-OES; iCAP 6300; Thermo Fisher Scientific). For pH measurements, a pH meter (Navi F-52; Horiba Instruments) was used. In order to prepare deionized water with a resistivity of > 18.2 MΩ cm, an Arium Pro water purification system (Sartorius Stedium Biotech GmbH) was used. A natural incubator (NIB-82; Iwaki Asahi Techno Glass) was used for heating.

### 2. Experimental details

#### 2.1. Synthetic experiments

##### 2.1.1. Synthesis of 3a–d (esterification)

After cellulose (1 equiv.) was dried for 2 h at 90 °C *in vacuo* (0.1 mmHg), DMA (5.4 mL mmol^−1^) was added, and the resultant slurry was stirred for 20 h at 90 °C. LiCl (*ca.* 7–8 equiv.) was added to the slurry precooled to room temperature, and the mixture was stirred for 1 h at room temperature. 2a–d (6 equiv.), DMAP (6 equiv.) and EDC-HCl (6 equiv.) were added to the resultant solution at 0 °C, and the mixture was stirred for 24 h at room temperature. The reaction mixture was poured into an excess amount of MeOH/H_2_O (70/30–80/20, v/v) under stirring. The formed precipitate was collected by centrifugation, washed with MeOH/H_2_O (80/20, v/v), and dried *in vacuo* to give 3a–d. The product was often contaminated with small amounts of waste based on reagents used in the reaction, but those could be used for the next step without further purification. For identification, the purer product could be obtained by repeating precipitation and washing with MeOH/H_2_O.

###### 3a

Cellulose (301 mg, 1.86 mmol), DMA (20 mL), LiCl (642 mg, 15.1 mmol), 2a (2.29 g, 11.3 mmol), DMAP (1.38 g, 11.3 mmol) and EDC-HCl (2.16 g, 11.3 mmol) were used for the reaction to give 3a (1.17 g, 84% yield) as a white solid. ^1^H NMR (500 MHz, CDCl_3_, 55 °C): *δ* 5.05 (br, 1H), 4.73 (br, 1H), 4.55 (br, 1H), 4.48 (br, 1H), 4.07 (br, 1H), 3.33–3.70 (m, 8H), 2.82–2.87 (m, 9H), 2.43–2.56 (m, 6H), 1.43 (s, 27H); IR (KBr, cm^−1^): 1745, 1691. Anal. calcd for C_33_H_55_N_3_O_14_·H_2_O: C, 53.87; H, 7.81; N, 5.71. Found: C, 54.12; H, 7.58; N, 5.70.

###### 3b

Cellulose (1.00 g, 6.17 mmol), DMA (31 mL), LiCl (1.96 g, 46.3 mmol), 2b (9.00 g, 37.0 mmol), DMAP (4.52 g, 37.0 mmol) and EDC-HCl (7.09 g, 37.0 mmol) were used for the reaction to give 3b (3.87 g, 75% yield) as a white solid. ^1^H NMR (500 MHz, CDCl_3_, 55 °C): *δ* 5.06 (br, 1H), 4.68 (br, 1H), 4.53 (br, 1H), 4.34 (br, 1H), 3.93–4.20 (m, 7H), 3.76 (br, 1H), 3.51 (br, 1H), 2.72 (br, 6H), 2.10–2.30 (m, 6H), 1.55–1.95 (br, 9H), 1.45 (s, 27H), 1.00–1.25 (m, 6H); IR (KBr, cm^−1^): 1747, 1691. Anal. calcd for C_42_H_67_N_3_O_14_: C, 60.20; H, 8.06; N, 5.01. Found: C, 59.81; H, 8.22; N, 5.04.

###### 3c

Cellulose (253 mg, 1.56 mmol), DMA (9.4 mL), LiCl (528 mg, 12.5 mmol), 2c (2.03 g, 9.43 mmol), DMAP (1.17 g, 9.56 mmol) and EDC-HCl (1.79 g, 9.35 mmol) were used for the reaction to give 3c (1.15 g, 98% yield) as a white solid. ^1^H NMR (500 MHz, CDCl_3_, 55 °C): *δ* 5.07 (br, 1H), 4.00–4.72 (m, 3H), 2.70–3.90 (m, 18H), 1.90–2.30 (m, 6H), 1.45 (s, 27H); IR (KBr, cm^−1^): 1751, 1691. Anal. calcd for C_36_H_55_N_3_O_14_·0.5H_2_O: C, 56.68; H, 7.40; N, 5.51. Found: C, 56.68; H, 7.33; N, 5.55.

###### 3d

Cellulose (1.25 g, 7.71 mmol), DMA (42 mL), LiCl (2.60 g, 61.4 mmol), 2d (10.0 g, 46.6 mmol), DMAP (5.72 g, 46.8 mmol) and EDC-HCl (9.00 g, 47.0 mmol) were used for the reaction to give 3d (4.82 g, 82% yield) as a white solid. ^1^H NMR (500 MHz, CDCl_3_, 55 °C): *δ* 3.20–5.20 (br, 16H), 1.58–2.40 (br, 12H), 1.43 (br, 27H); IR (KBr, cm^−1^): 1755, 1709. Anal. calcd for C_36_H_55_N_3_O_14_·H_2_O: C, 56.02; H, 7.44; N, 5.44. Found: C, 55.92; H, 7.31; N, 5.45.

##### 2.1.2. Synthesis of 4a–d (deprotection)

TFA (5 mL mmol^−1^) was added to 3a–d, and the mixture was stirred for 7 h at room temperature. The reaction mixture was poured into an excess amount of Et_2_O under stirring. The formed precipitate was collected by centrifugation, washed with Et_2_O, and dried *in vacuo* to give 4a–d.

###### 4a

TFA (7.8 mL) and 3a (1.17 g, 1.63 mmol) were used for the reaction to give 4a (1.08 g, 92% yield) as a white solid. ^1^H NMR (500 MHz, D_2_O, 80 °C): *δ* 5.71 (br, 1H), 4.10–5.40 (m, 6H, partially overlapped with a water signal), 3.85 (br, 6H), 3.20–3.55 (m, 15H), the signal based on NH_2_ was not observed due to H-D exchange; IR (KBr, cm^−1^): 1750, 1681. Anal. calcd for C_24_H_34_N_3_O_14_F_9_·2H_2_O: C, 36.23; H, 4.81; N, 5.28. Found: C, 36.32; H, 4.43; N, 5.22.

###### 4b

TFA (8.0 mL) and 3b (1.36 g, 1.62 mmol) were used for the reaction to give 4b (1.37 g, 96% yield) as a white solid. ^1^H NMR (500 MHz, D_2_O): *δ* 5.09 (br, 1H), 4.30–4.80 (m, 5H, partially overlapped with a water signal), 3.75 (br, 1H), 3.42 (br, 6H), 2.98 (br, 6H), 1.80–2.50 (m, 15H), 1.30–1.50 (m, 6H), the signal based on NH_2_ was not observed due to H-D exchange; IR (KBr, cm^−1^): 1745, 1689. Anal. calcd for C_33_H_46_N_3_O_14_F_9_·2H_2_O: C, 43.28; H, 5.50; N, 4.59. Found: C, 42.84; H, 5.07; N, 4.51.

###### 4c

TFA (32 mL) and 3c (4.80 g, 6.37 mmol) were used for the reaction to give 4c (4.73 g, 93% yield) as a white solid. ^1^H NMR (500 MHz, D_2_O): *δ* 5.19 (br, 1H), 3.303.80–4.80 (m, 6H, partially overlapped with a water signal), 3.30–3.70 (m, 12H), 1.70–2.50 (m, 9H), the signal based on NH_2_ was not observed due to H-D exchange; IR (KBr, cm^−1^): 1747, 1679. Anal. calcd for C_27_H_34_N_3_O_14_F_9_·H_2_O: C, 39.86; H, 4.46; N, 5.16. Found: C, 39.49; H, 4.28; N, 5.15.

###### 4d

TFA (32 mL) and 3d (4.82 g, 6.40 mmol) were used for the reaction to give 4d (4.09 g, 80% yield) as a white solid. ^1^H NMR (500 MHz, D_2_O): *δ* 5.42 (br, 1H), 4.55–5.20 (m, 5H, partially overlapped with a water signal), 3.80–4.40 (m, 4H), 3.50 (br, 6H), 1.85–2.70 (m, 12H), the signal based on NH_2_ was not observed due to H-D exchange; IR (KBr, cm^−1^): 1755, 1678. Anal. calcd for C_27_H_34_N_3_O_14_F_9_·H_2_O: C, 39.86; H, 4.46; N, 5.16. Found: C, 39.55; H, 4.54; N, 4.97.

##### 2.1.3. Synthesis of 5a–d (dithiocarbamation)

To a solution of 4a–d in DMSO (5 mL mmol^−1^) was added CS_2_ (15 equiv.) and 10% Me_4_NOH solution in MeOH (*ca.* 6–7 equiv.) at 0 °C, and the resultant suspension was stirred at room temperature. The reaction mixture became almost homogeneous in 7–48 h. The reaction mixture was poured into an excess amount of MeOH (for 4a and 4b) or EtOH (for 4c and 4d) under stirring. The formed precipitate was collected by centrifugation, washed with MeOH (for 4a and 4b) or EtOH (for 4c and 4d), and dried *in vacuo* to give 5a–d.

###### 5a

4a (1.55 g, 2.04 mmol), DMSO (10 mL), CS_2_ (1.84 mL, 30.5 mmol) and 10% Me_4_NOH solution in MeOH (14 mL, 13.9 mmol) were used for the reaction (7 h) to give 5a (605 mg, 34% yield) as a white solid. ^1^H NMR (500 MHz, D_2_O): *δ* 3.90–5.50 (m, 7H, partially overlapped with a water signal), 3.45 (br, 6H), 3.18 (br, 9H), 3.16 (s, 36H); IR (KBr, cm^−1^): 1741, 1485, 1156. The value observed by elemental analysis was significantly different from the theoretical value because the sample contained a significant amount of water or because the side chain was partially degraded by hydrolysis (for example, Anal. calcd for C_33_H_64_N_6_O_8_S_6_: C, 45.81; H, 7.46; N, 9.71. Found: C, 41.42; H, 7.13; N, 8.14).

###### 5b

4b (507 mg, 0.576 mmol), DMSO (2.8 mL), CS_2_ (0.52 mL, 8.61 mmol) and 10% Me_4_NOH solution in MeOH (3.7 mL, 3.66 mmol) were used for the reaction (48 h) to give 5b (496 mg, 87% yield) as a white solid. ^1^H NMR (500 MHz, CD_3_CO_2_D): *δ* 3.70–5.50 (m, 7H), 3.54 (br, 6H), 3.22 (s, 36H), 3.09 (br, 6H), 1.80–2.50 (br, 15H, partially overlapped with a solvent signal), 1.40–1.70 (m, 6H); IR (KBr, cm^−1^): 1741, 1410, 1174. The value observed by elemental analysis was significantly different from the theoretical value because the sample contained a significant amount of water or because the side chain was partially degraded by hydrolysis (for example, Anal. calcd for C_42_H_76_N_6_O_8_S_6_: C, 51.19; H, 7.77; N, 8.53. Found: C, 47.76; H, 6.69; N, 6.43).

###### 5c

4c (421 mg, 0.529 mmol), DMSO (2.7 mL), CS_2_ (0.49 mL, 8.12 mmol) and 10% Me_4_NOH solution in MeOH (3.7 mL, 3.66 mmol) were used for the reaction (48 h) to give 5c (404 mg, 83% yield) as a white solid. ^1^H NMR (500 MHz, D_2_O): *δ* 5.27 (br, 1H), 3.30–5.00 (m, 18H, partially overlapped with a water signal), 3.20 (s, 36H), 1.90–2.70 (br, 9H).; IR (KBr, cm^−1^): 1740, 1384, 1165. The value observed by elemental analysis was significantly different from the theoretical value because the sample contained a significant amount of water or because the side chain was partially degraded by hydrolysis (for example, Anal. calcd for C_36_H_64_N_6_O_8_S_6_: C, 47.97; H, 7.15; N, 9.32. Found: C, 44.32; H, 7.34; N, 8.51).

###### 5d

7 h: 4d (1.00 g, 1.26 mmol), DMSO (6.3 mL), CS_2_ (1.14 mL, 18.9 mmol) and 10% Me_4_NOH solution in MeOH (8.7 mL, 8.61 mmol) were used for the reaction (7 h) to give 5d (915 mg, 81% yield) as a white solid. 24 h: 4d (504 mg, 0.633 mmol), DMSO (3.1 mL), CS_2_ (0.57 mL, 9.44 mmol) and 10% Me_4_NOH solution in MeOH (4.3 mL, 4.27 mmol) were used for the reaction (24 h) to give 5d (519 mg, 91% yield) as a white solid. ^1^H NMR (500 MHz, D_2_O): *δ* 5.04 (br, 3H, partially overlapped with a water signal), 3.90 (br, 6H), 3.17 (s, 36H), 1.70–2.60 (br, 12H), signals based on the cellulose moiety (7H) were obscure due to broadening; IR (KBr, cm^−1^): 1737, 1375, 1183. The value observed by elemental analysis was significantly different from the theoretical value because the sample contained a significant amount of water or because the side chain was partially degraded by hydrolysis (for example, Anal. calcd for C_36_H_64_N_6_O_8_S_6_: C, 47.97; H, 7.15; N, 9.32. Found: C, 42.50; H, 7.40; N, 7.81).

#### 2.2. Batch sorption experiments

The durability of the sorbents was investigated by comparing the sorption capacities of As(iii) before and after keeping them at 40 °C for fortnight. Sorption tests were conducted in 50 mL centrifuge tubes containing 0.02 g of sorbent and 10 mL of 2 mmol L^−1^ As(iii) solutions by agitating the tubes for 20 minutes at 25 °C and 200 rpm. Then, the solutions were collected by filtrating suspensions through a 0.45 μm membrane filter. Subsequently, the metal concentrations in the solutions were determined with ICP-OES. The sorption capacities of As(iii) (*q*_e_) were calculated according to the equation shown below:1
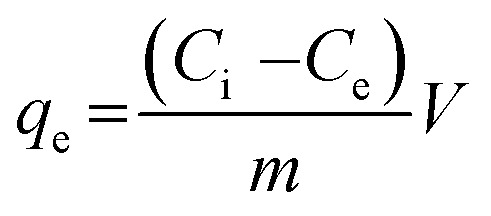
where *q*_e_ (μmol g^−1^) represents the sorption capacity of As(iii), *C*_i_ and *C*_e_ (μmol L^−1^) refer to initial and equilibrium concentrations of As(iii), *m* (g) is the weight of the sorbent, and *V* (L) is the solution volume.

The removal efficiency of various elements was examined using polymer 5d. The solution containing 21 elements was prepared by diluting the desired amount of the ICP multi-element standard solution with deionized water so that the concentrations became 5 mg L^−1^. Then, the pH was adjusted to pH 3 using 0.1 mol L^−1^ HNO_3_ or NaOH solution, and sorption tests were carried out according to the procedure mentioned above. The removed metal percentages (%) were calculated from the following equation:2
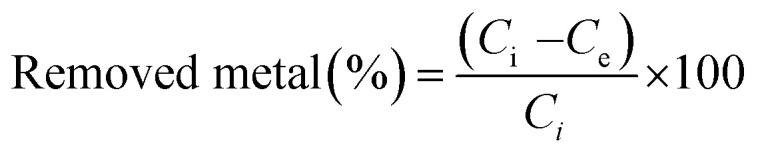


## Conflicts of interest

The authors declare the following conflict of interest(s): Kanazawa University and Daicel Corporation hold or have a filed patent related to this work (Patent Application No. PCT/JP2020/21903).

## Supplementary Material

RA-010-D0RA05573E-s001
