# Supplementary material for: Dithiocarbamate-modified cellulose-based sorbents with high storage stability for selective removal of arsenite and hazardous heavy metals
Source: RSC Adv. 2020 Aug 17;10(50):30238–44. doi: 10.1039/d0ra05573e (PMC9056302; doi:10.1039/d0ra05573e)
Supplement: RA-010-D0RA05573E-s001 [file RA-010-D0RA05573E-s001.pdf]

Electronic Supplementary Material (ESI) for RSC Advances.
This journal is © The Royal Society of Chemistry 2020

# Electronic Supplementary Information

## Dithiocarbamate-modified cellulose-based sorbents with high storage stability for selective removal of arsenite and hazardous heavy metals

Futo Morita,[†a] Keisuke Nakakubo,[†a] Koki Yunoshita,[†a] Masaru Endo,[a,b] Foni B. Biswas,[a,c] Tatsuya Nishimura,[a] Asami S. Mashio,[a] Hiroshi Hasegawa,*[a] Tsuyoshi Taniguchi,*[a] Katsuhiro Maeda*[a,d]

[a]*Graduate School of Natural Science and Technology, Kanazawa University, Kakuma-machi, Kanazawa 920-1192, Japan.*

[b]*Daicel Corporation, 1239 Shinzaike, Aboshi-ku, Himeji, Hyogo 671-1283, Japan.*

[c]*Department of Chemistry, University of Chittagong, Chittagong 4331, Bangladesh.*

[d]*Nano Life Science Institute (WPI-NanoLSI), Kanazawa University, Kakuma-machi, Kanazawa 920-1192, Japan.*

*E-mail: hhiroshi@se.kanazawa-u.ac.jp, tsuyoshi@p.kanazawa-u.ac.jp, maeda@se.kanazawa-u.ac.jp*

†These authors contributed equally.

## Table of Contents

1. The result of batch sorption experiments for various metals........................................................S2

2. IR Spectra of DTC compounds…………….………………………..….………………......…...S3

3. References………………………………………………………..….………………..…………...…..S7

4. Copies of NMR spectra of synthesized compounds……………………...…………………..…...S8

# 1. The result of batch sorption experiments for various metals

| Metal | Removal (%) | Metal | Removal (%) | Metal | Removal (%) |
|---|---|---|---|---|---|
| Be(II) | 2.0 ± 1.5 | Al(III) | 0 | Cu(II) | 99.6 ± 0.01 |
| Mg(II) | 0 | Ti(IV) | 95.4 ± 0.1 | Zn(II) | 99.7 ± 0.04 |
| Ca(II) | 0 | V(IV) | 95.9 ± 1.6 | Ga(III) | 93.1 ± 1.2 |
| Sr(II) | 3.3 ± 0.4 | Mn(III) | 13.5 ± 0.9 | Cd(II) | 99.5 ± 0.02 |
| Ba(II) | 1.6 ± 0.6 | Fe(III) | 97.0 ± 0.5 | In(III) | 100 ± 0.09 |
| Sc(III) | 3.5 ± 0.6 | Co(II) | 100 ± 0.1 | Pb(II) | 100 ± 0.8 |
| Y(III) | 3.0 ± 0.3 | Ni(II) | 99.6 ± 0.04 | Bi(III) | 94.0 ± 0.6 |

# 2. IR spectra of DTC compounds

## 2-1. DTC compound 1 (previously reported)[1]

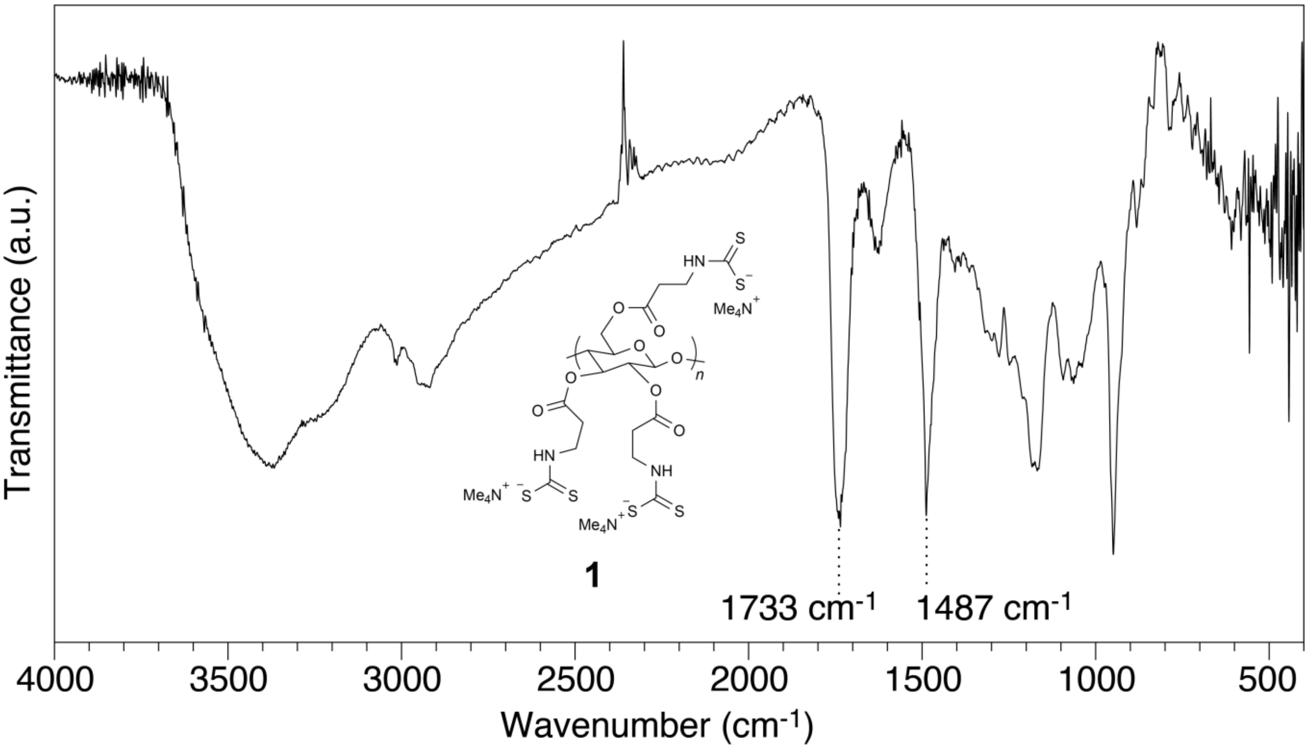


## 2-2. DTC compounds 5a–d

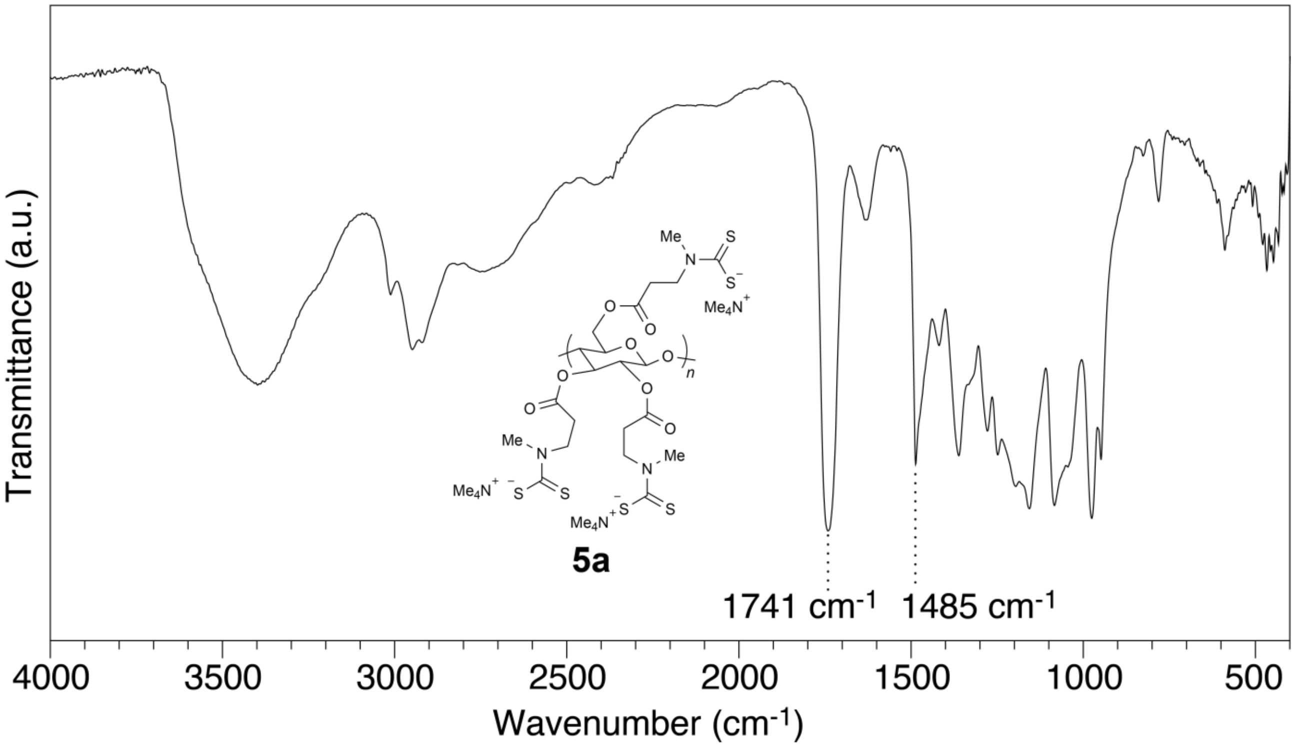

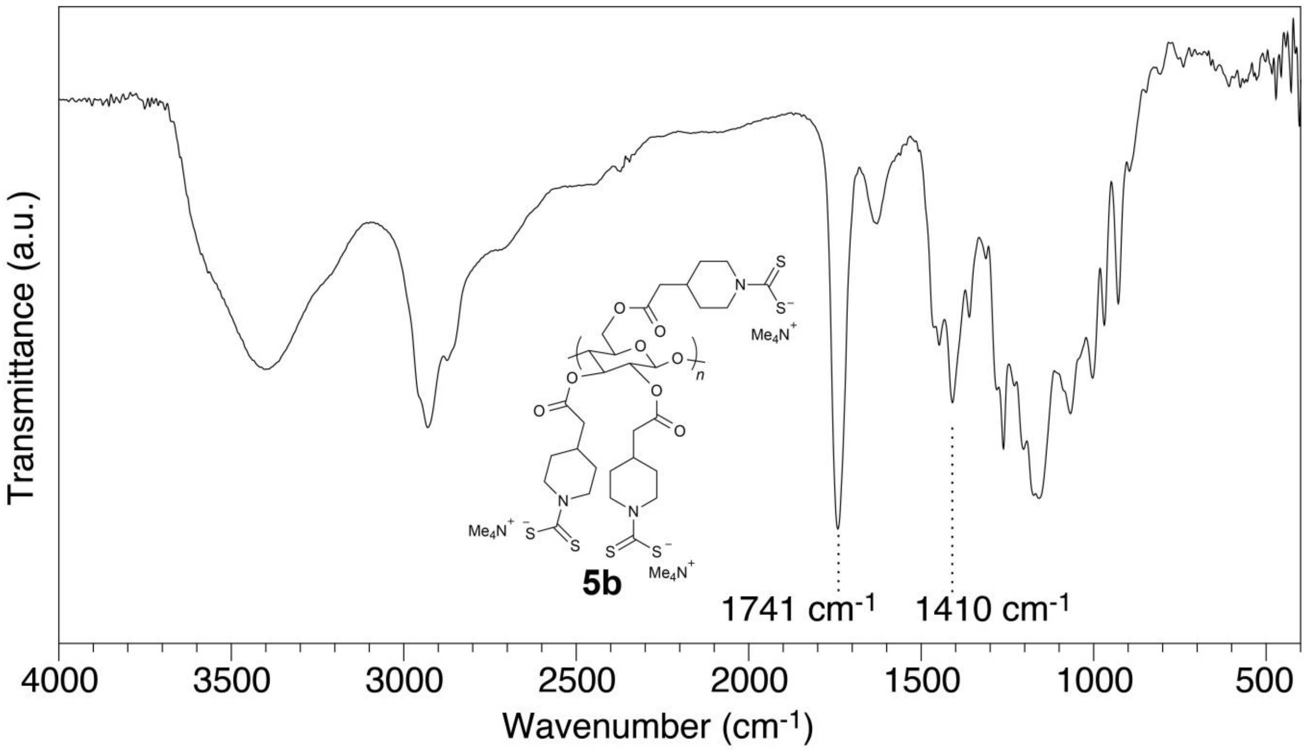

Transmittance (a.u.)
Me4N+
5b
1741 cm-1
1410 cm-1
4000
3500
3000
2500
2000
1500
1000
500
Wavenumber (cm-1)


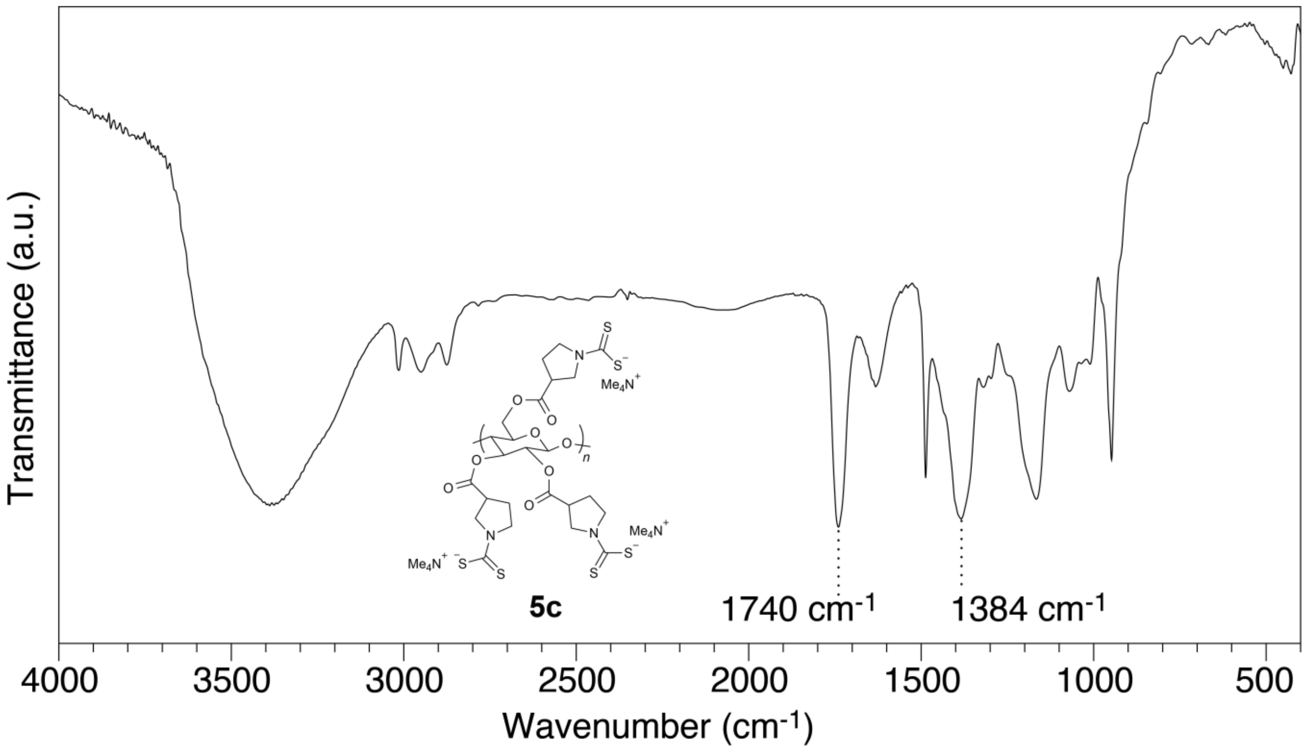

Transmittance (a.u.)
Me4N+
5c
1740 cm-1
1384 cm-1
4000
3500
3000
2500
2000
1500
1000
500
Wavenumber (cm-1)

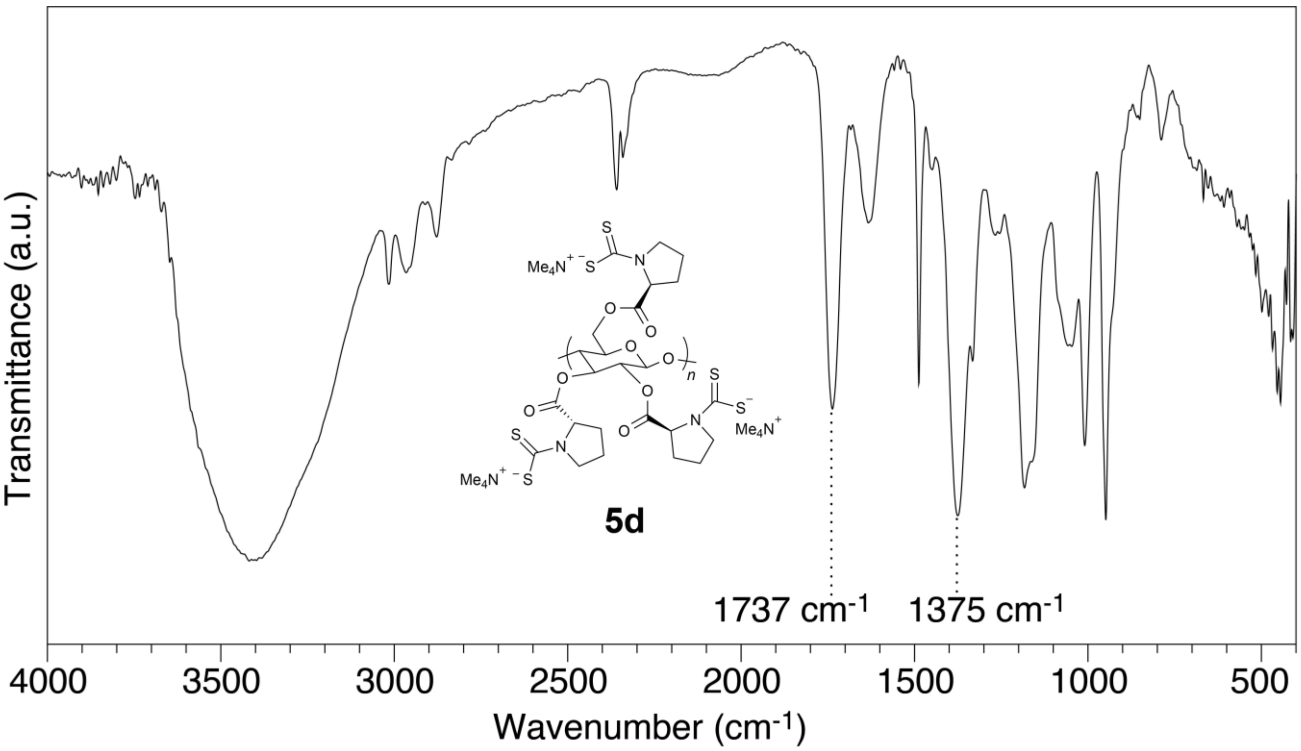

Transmittance (a.u.)
Wavenumber (cm-1)
4000
3500
3000
2500
2000
1500
1000
500
1737 cm-1
1375 cm-1
5d

## 2-3. IR spectra of 1 and 5d after a storage at 40 °C for 2 weeks under air

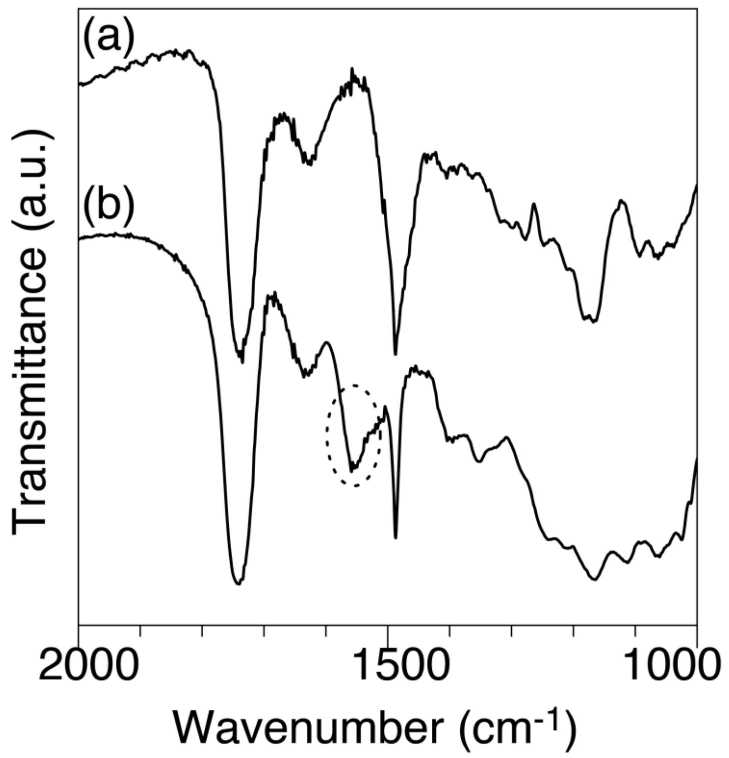


**Fig. S1.** IR spectrum of DTC compound **1** (a) and a new chracteristic signal appeared at around 1560 $cm^{-1}$ after 2 weeks (b).

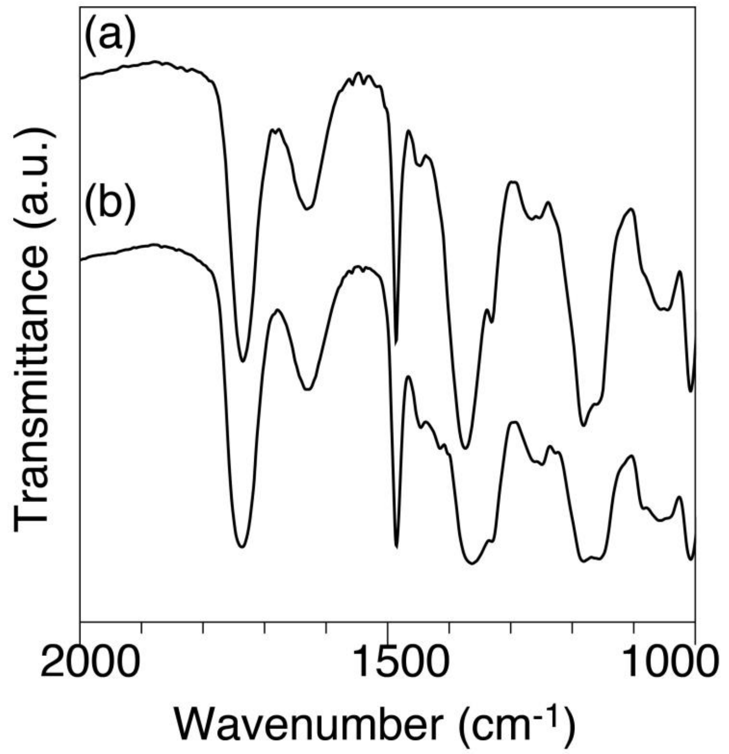


**Fig. S2.** IR spectrum of DTC compound **5d** (a) and the spectrum hardly changed after 2 weeks (b).

## 3. References

1. K. Nakakubo, H. Hasegawa, M. Ito, K. Yamazaki, M. Miyaguchi, F. B. Biswas, T. Ikai and K. Maeda, *J. Hazard. Mater.,* 2019, **380**, 120816.

## 4. Copies of NMR spectra of synthesized compounds

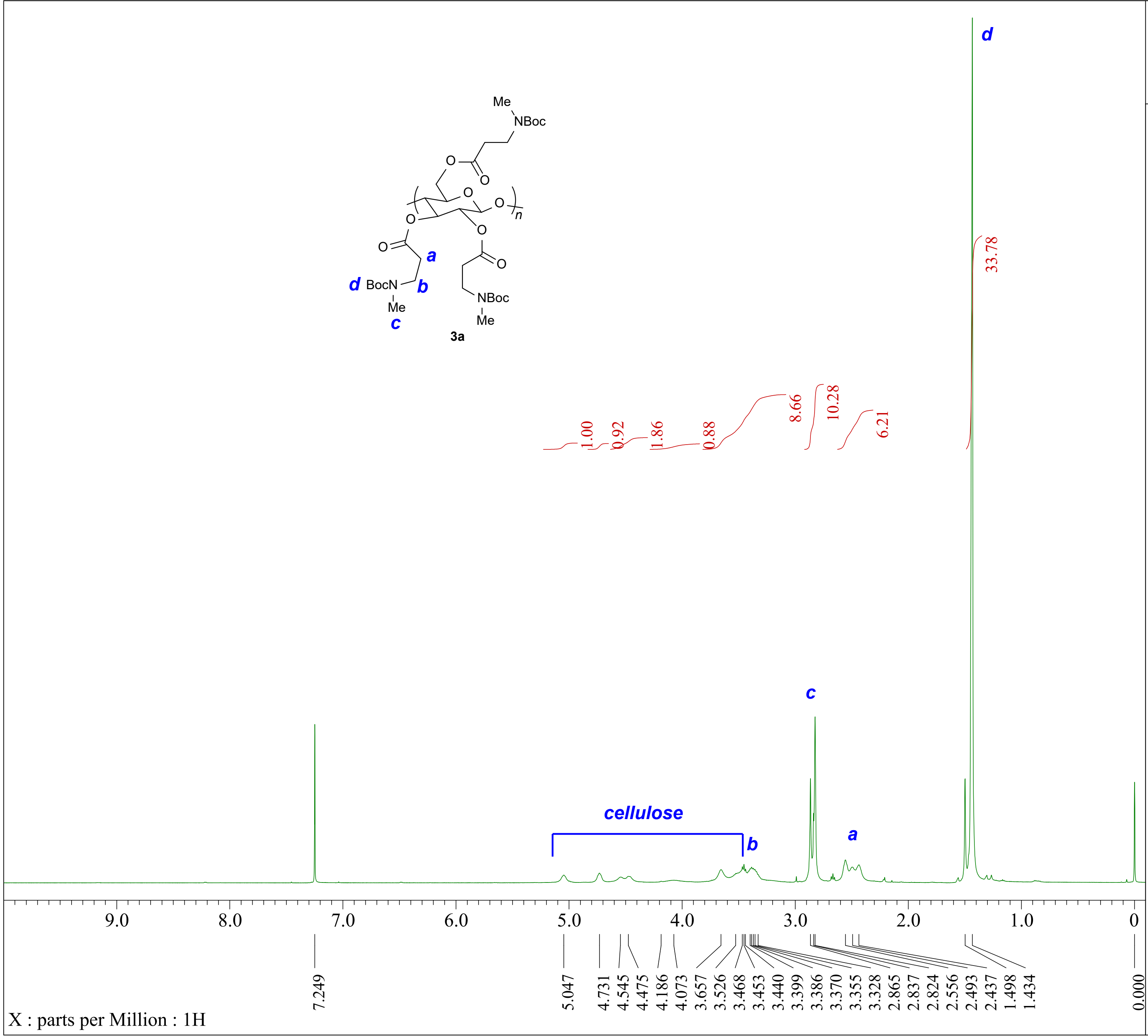


JEOL Solutions for Innovation

```
Filename          = FM98-cel-2mnm-boc-4.jdf
Author            = delta
Experiment        = single_pulse.ex2
Sample_Id         = S#415097
Solvent           = CHLOROFORM-D
Creation_Time     = 20-JAN-2020 11:35:39
Revision_Time     =  4-JUN-2020 10:41:58
Current_Time      =  4-JUN-2020 10:43:02

Comment           = FM98-cel-2mnm-boc
Data_Format       = 1D COMPLEX
Dim_Size          = 13107
Dim_Title         = 1H
Dim_Units         = [ppm]
Dimensions        = X
Site              = ECA 500
Spectrometer      = JNM-ECA500

Field_Strength    = 11.7473579[T] (500[MHz])
X_Acq_Duration    = 1.74587904[s]
X_Domain          = 1H
X_Freq            = 500.15991521[MHz]
X_Offset          = 5.0[ppm]
X_Points          = 16384
X_Prescans        = 1
X_Resolution      = 0.57277737[Hz]
X_Sweep           = 9.38438438[kHz]
Irr_Domain        = 1H
Irr_Freq          = 500.15991521[MHz]
Irr_Offset        = 5.0[ppm]
Tri_Domain        = 1H
Tri_Freq          = 500.15991521[MHz]
Tri_Offset        = 5.0[ppm]
Clipped           = FALSE
Scans             = 256
Total_Scans       = 256

Relaxation_Delay  = 2[s]
Recvr_Gain        = 54
Temp_Get          = 55[dC]
X_90_Width        = 11.6[us]
X_Acq_Time        = 1.74587904[s]
X_Angle           = 45[deg]
X_Atn             = 3.6[dB]
X_Pulse           = 5.8[us]
Irr_Mode          = Off
Tri_Mode          = Off
Dante_Presat      = FALSE
Initial_Wait      = 1[s]
Repetition_Time   = 3.74587904[s]
```

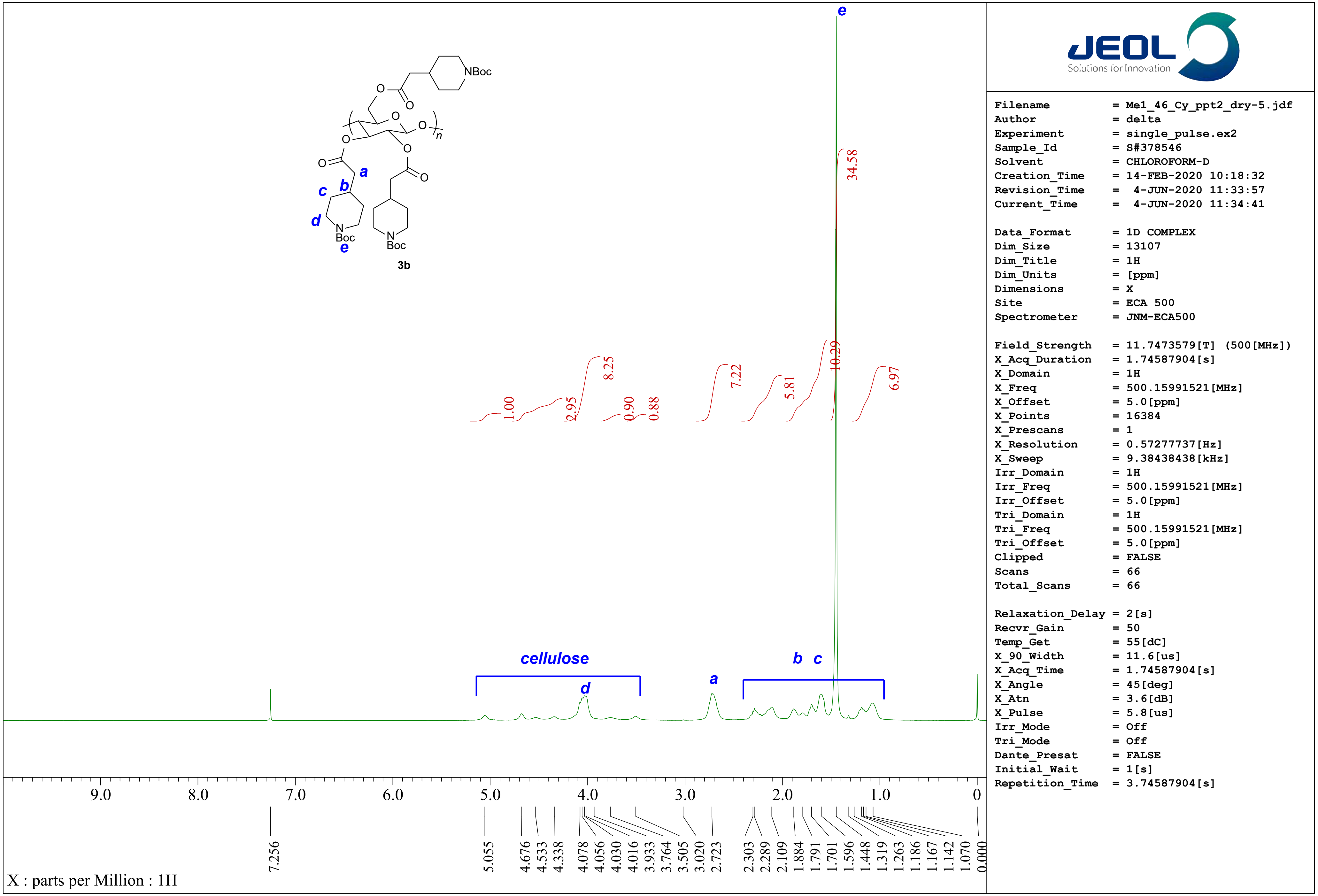
NBoc
O
O
O
O
O
O
O
n
O
a
b
c
d
N
Boc
e
N
Boc
3b
e
34.58
1.00
2.95
8.25
0.90
0.88
7.22
5.81
10.29
6.97
cellulose
d
a
b c
9.0
8.0
7.0
6.0
5.0
4.0
3.0
2.0
1.0
0
7.256
5.055
4.676
4.533
4.338
4.078
4.056
4.030
4.016
3.933
3.764
3.505
3.020
2.723
2.303
2.289
2.109
1.884
1.791
1.701
1.596
1.448
1.319
1.263
1.186
1.167
1.142
1.070
0.000
X : parts per Million : 1H
JEOL
Solutions for Innovation
Filename = Me1_46_Cy_ppt2_dry-5.jdf
Author = delta
Experiment = single_pulse.ex2
Sample_Id = S#378546
Solvent = CHLOROFORM-D
Creation_Time = 14-FEB-2020 10:18:32
Revision_Time = 4-JUN-2020 11:33:57
Current_Time = 4-JUN-2020 11:34:41
Data_Format = 1D COMPLEX
Dim_Size = 13107
Dim_Title = 1H
Dim_Units = [ppm]
Dimensions = X
Site = ECA 500
Spectrometer = JNM-ECA500
Field_Strength = 11.7473579[T] (500[MHz])
X_Acq_Duration = 1.74587904[s]
X_Domain = 1H
X_Freq = 500.15991521[MHz]
X_Offset = 5.0[ppm]
X_Points = 16384
X_Prescans = 1
X_Resolution = 0.57277737[Hz]
X_Sweep = 9.38438438[kHz]
Irr_Domain = 1H
Irr_Freq = 500.15991521[MHz]
Irr_Offset = 5.0[ppm]
Tri_Domain = 1H
Tri_Freq = 500.15991521[MHz]
Tri_Offset = 5.0[ppm]
Clipped = FALSE
Scans = 66
Total_Scans = 66
Relaxation_Delay = 2[s]
Recvr_Gain = 50
Temp_Get = 55[dC]
X_90_Width = 11.6[us]
X_Acq_Time = 1.74587904[s]
X_Angle = 45[deg]
X_Atn = 3.6[dB]
X_Pulse = 5.8[us]
Irr_Mode = Off
Tri_Mode = Off
Dante_Presat = FALSE
Initial_Wait = 1[s]
Repetition_Time = 3.74587904[s]

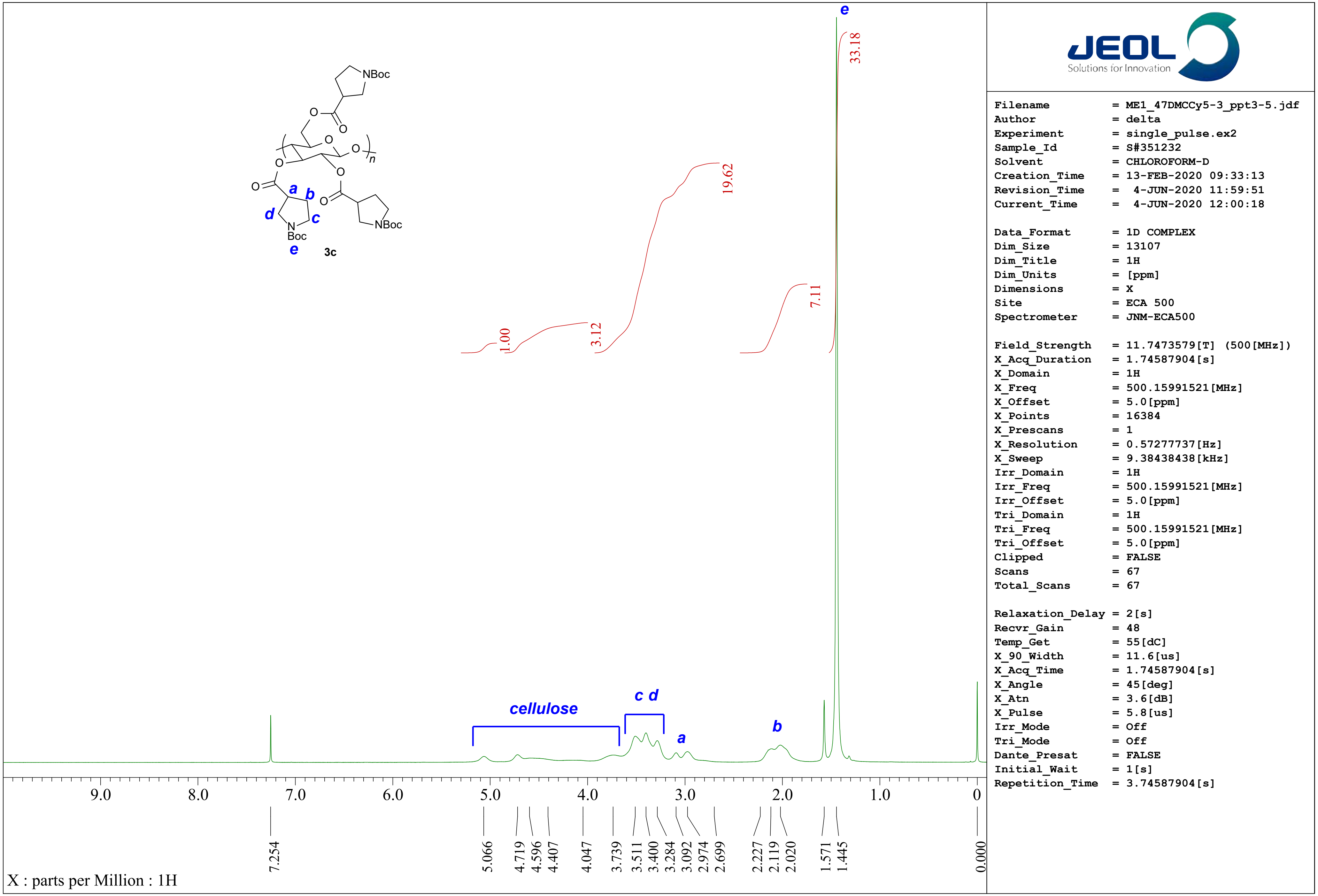

NBoc
O
O
O
O
O
n
O
O
a
b
d
c
N
Boc
e
O
O
NBoc
3c
e
33.18
19.62
7.11
1.00
3.12
cellulose
c d
a
b
9.0
8.0
7.0
6.0
5.0
4.0
3.0
2.0
1.0
0
7.254
5.066
4.719
4.596
4.407
4.047
3.739
3.511
3.400
3.284
3.092
2.974
2.699
2.227
2.119
2.020
1.571
1.445
0.000
X : parts per Million : 1H
JEOL
Solutions for Innovation
Filename = ME1_47DMCCy5-3_ppt3-5.jdf
Author = delta
Experiment = single_pulse.ex2
Sample_Id = S#351232
Solvent = CHLOROFORM-D
Creation_Time = 13-FEB-2020 09:33:13
Revision_Time = 4-JUN-2020 11:59:51
Current_Time = 4-JUN-2020 12:00:18
Data_Format = 1D COMPLEX
Dim_Size = 13107
Dim_Title = 1H
Dim_Units = [ppm]
Dimensions = X
Site = ECA 500
Spectrometer = JNM-ECA500
Field_Strength = 11.7473579[T] (500[MHz])
X_Acq_Duration = 1.74587904[s]
X_Domain = 1H
X_Freq = 500.15991521[MHz]
X_Offset = 5.0[ppm]
X_Points = 16384
X_Prescans = 1
X_Resolution = 0.57277737[Hz]
X_Sweep = 9.38438438[kHz]
Irr_Domain = 1H
Irr_Freq = 500.15991521[MHz]
Irr_Offset = 5.0[ppm]
Tri_Domain = 1H
Tri_Freq = 500.15991521[MHz]
Tri_Offset = 5.0[ppm]
Clipped = FALSE
Scans = 67
Total_Scans = 67
Relaxation_Delay = 2[s]
Recvr_Gain = 48
Temp_Get = 55[dC]
X_90_Width = 11.6[us]
X_Acq_Time = 1.74587904[s]
X_Angle = 45[deg]
X_Atn = 3.6[dB]
X_Pulse = 5.8[us]
Irr_Mode = Off
Tri_Mode = Off
Dante_Presat = FALSE
Initial_Wait = 1[s]
Repetition_Time = 3.74587904[s]

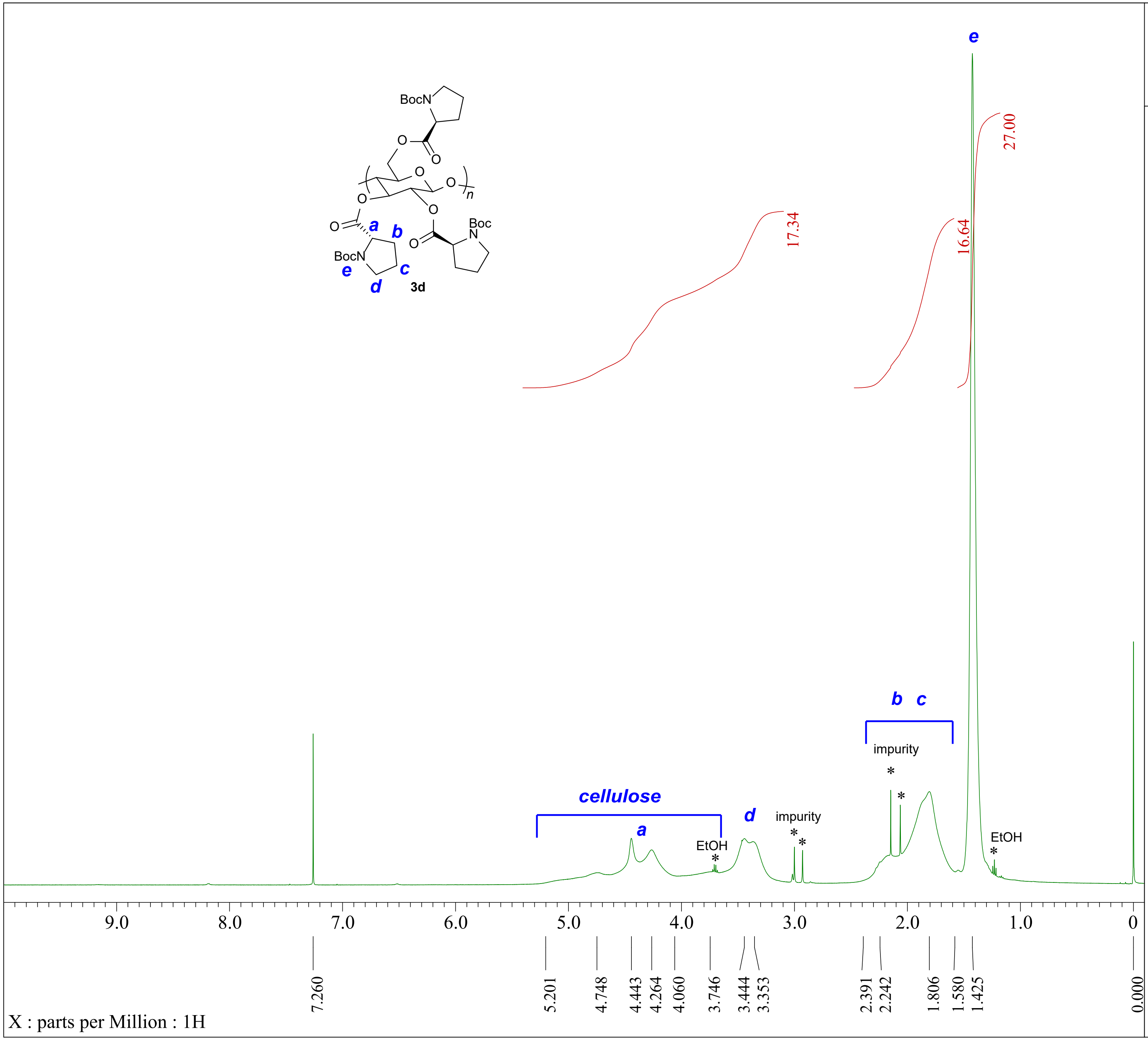


JEOL
Solutions for Innovation

```
Filename           = ME1_44_ppt3-5.jdf
Author             = delta
Experiment         = single_pulse.ex2
Sample_Id          = S#685130
Solvent            = CHLOROFORM-D
Creation_Time      =  6-FEB-2020 18:54:53
Revision_Time      =  4-JUN-2020 12:15:47
Current_Time       =  4-JUN-2020 12:16:47

Data_Format        = 1D COMPLEX
Dim_Size           = 13107
Dim_Title          = 1H
Dim_Units          = [ppm]
Dimensions         = X
Site               = ECA 500
Spectrometer       = JNM-ECA500

Field_Strength     = 11.7473579[T] (500[MHz])
X_Acq_Duration     = 1.74587904[s]
X_Domain           = 1H
X_Freq             = 500.15991521[MHz]
X_Offset           = 5.0[ppm]
X_Points           = 16384
X_Prescans         = 1
X_Resolution       = 0.57277737[Hz]
X_Sweep            = 9.38438438[kHz]
Irr_Domain         = 1H
Irr_Freq           = 500.15991521[MHz]
Irr_Offset         = 5.0[ppm]
Tri_Domain         = 1H
Tri_Freq           = 500.15991521[MHz]
Tri_Offset         = 5.0[ppm]
Clipped            = FALSE
Scans              = 80
Total_Scans        = 80

Relaxation_Delay   = 2[s]
Recvr_Gain         = 44
Temp_Get           = 55[dC]
X_90_Width         = 11.6[us]
X_Acq_Time         = 1.74587904[s]
X_Angle            = 45[deg]
X_Atn              = 3.6[dB]
X_Pulse            = 5.8[us]
Irr_Mode           = Off
Tri_Mode           = Off
Dante_Presat       = FALSE
Initial_Wait       = 1[s]
Repetition_Time    = 3.74587904[s]
```

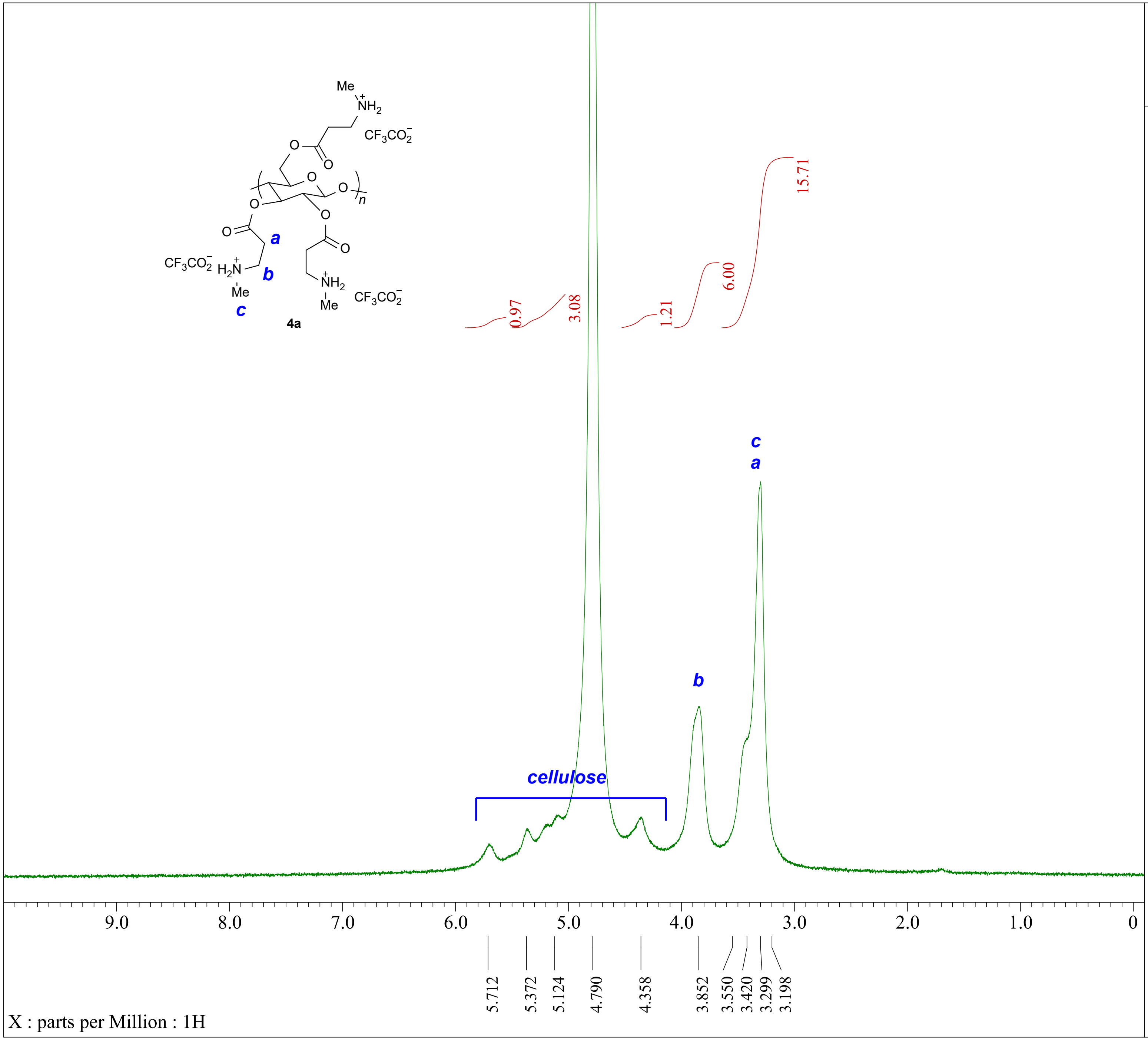


| | |
|---|---|
| Filename | = FM113-cel-2mnm-tfa-4.jdf |
| Author | = delta |
| Experiment | = single_pulse.ex2 |
| Sample_Id | = S#437940 |
| Solvent | = D2O |
| Creation_Time | = 13-FEB-2020 12:09:32 |
| Revision_Time | = 4-JUN-2020 13:33:17 |
| Current_Time | = 4-JUN-2020 13:33:47 |
| Data_Format | = 1D COMPLEX |
| Dim_Size | = 13107 |
| Dim_Title | = 1H |
| Dim_Units | = [ppm] |
| Dimensions | = X |
| Site | = ECA 500 |
| Spectrometer | = JNM-ECA500 |
| Field_Strength | = 11.7473579[T] (500[MHz]) |
| X_Acq_Duration | = 1.74587904[s] |
| X_Domain | = 1H |
| X_Freq | = 500.15991521[MHz] |
| X_Offset | = 5.0[ppm] |
| X_Points | = 16384 |
| X_Prescans | = 1 |
| X_Resolution | = 0.57277737[Hz] |
| X_Sweep | = 9.38438438[kHz] |
| Irr_Domain | = 1H |
| Irr_Freq | = 500.15991521[MHz] |
| Irr_Offset | = 5.0[ppm] |
| Tri_Domain | = 1H |
| Tri_Freq | = 500.15991521[MHz] |
| Tri_Offset | = 5.0[ppm] |
| Clipped | = FALSE |
| Scans | = 256 |
| Total_Scans | = 256 |
| Relaxation_Delay | = 2[s] |
| Recvr_Gain | = 66 |
| Temp_Get | = 80[dC] |
| X_90_Width | = 11.6[us] |
| X_Acq_Time | = 1.74587904[s] |
| X_Angle | = 45[deg] |
| X_Atn | = 3.6[dB] |
| X_Pulse | = 5.8[us] |
| Irr_Mode | = Off |
| Tri_Mode | = Off |
| Dante_Presat | = FALSE |
| Initial_Wait | = 1[s] |
| Repetition_Time | = 3.74587904[s] |

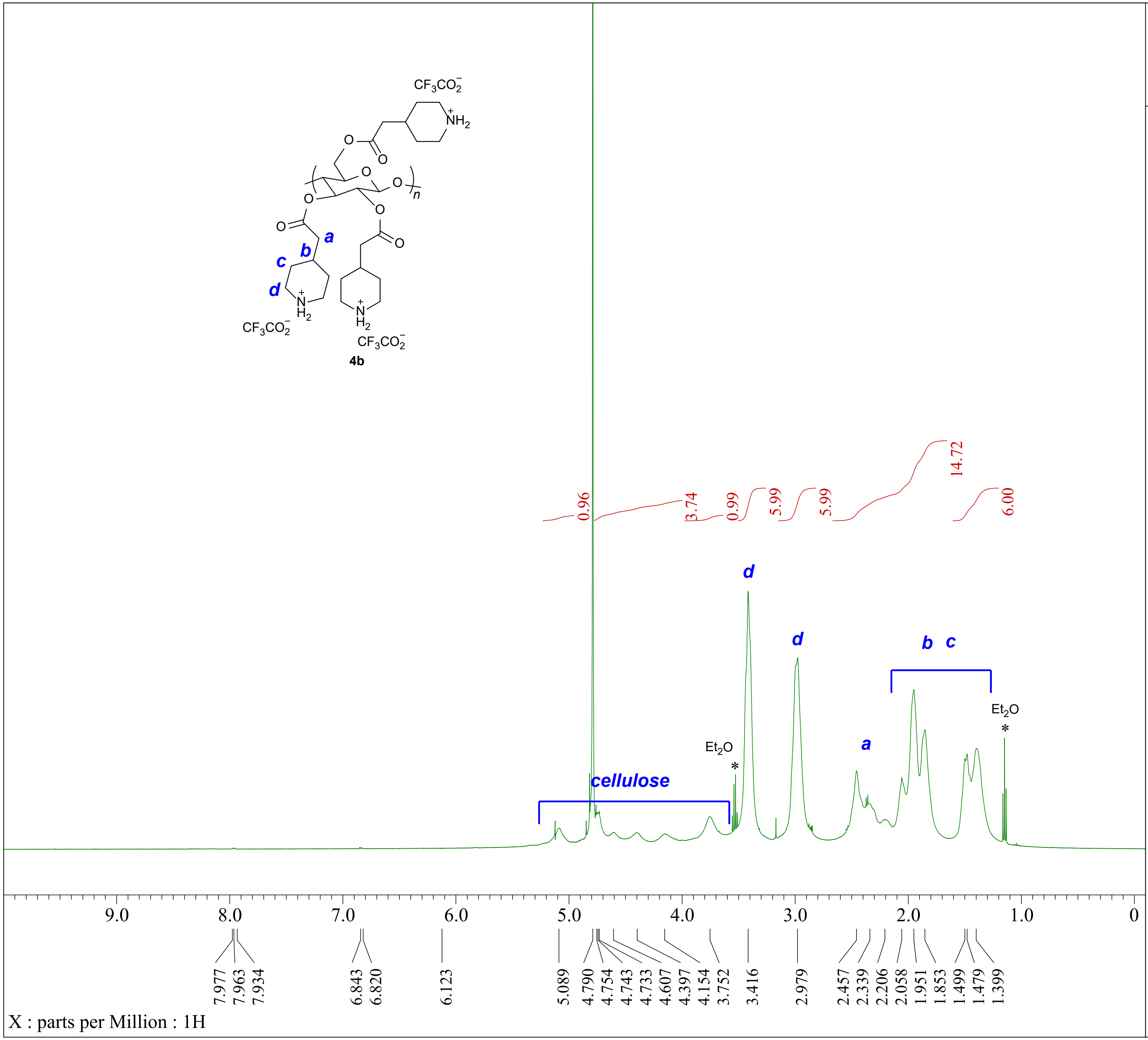


JEOL Solutions for Innovation

```
Filename          = ME1_29_recry_200413_D2O-5.
Author            = delta
Experiment        = single_pulse.ex2
Sample_Id         = S#348941
Solvent           = D2O
Creation_Time     = 13-APR-2020 09:27:40
Revision_Time     =  4-JUN-2020 13:54:29
Current_Time      =  4-JUN-2020 13:55:12

Data_Format       = 1D COMPLEX
Dim_Size          = 13107
Dim_Title         = 1H
Dim_Units         = [ppm]
Dimensions        = X
Site              = ECA 500
Spectrometer      = JNM-ECA500

Field_Strength    = 11.7473579[T] (500[MHz])
X_Acq_Duration    = 1.74587904[s]
X_Domain          = 1H
X_Freq            = 500.15991521[MHz]
X_Offset          = 5.0[ppm]
X_Points          = 16384
X_Prescans        = 1
X_Resolution      = 0.57277737[Hz]
X_Sweep           = 9.38438438[kHz]
Irr_Domain        = 1H
Irr_Freq          = 500.15991521[MHz]
Irr_Offset        = 5.0[ppm]
Tri_Domain        = 1H
Tri_Freq          = 500.15991521[MHz]
Tri_Offset        = 5.0[ppm]
Clipped           = FALSE
Scans             = 64
Total_Scans       = 64

Relaxation_Delay  = 2[s]
Recvr_Gain        = 40
Temp_Get          = 19.4[dC]
X_90_Width        = 11.4[us]
X_Acq_Time        = 1.74587904[s]
X_Angle           = 45[deg]
X_Atn             = 3.6[dB]
X_Pulse           = 5.7[us]
Irr_Mode          = Off
Tri_Mode          = Off
Dante_Presat      = FALSE
Initial_Wait      = 1[s]
Repetition_Time   = 3.74587904[s]
```

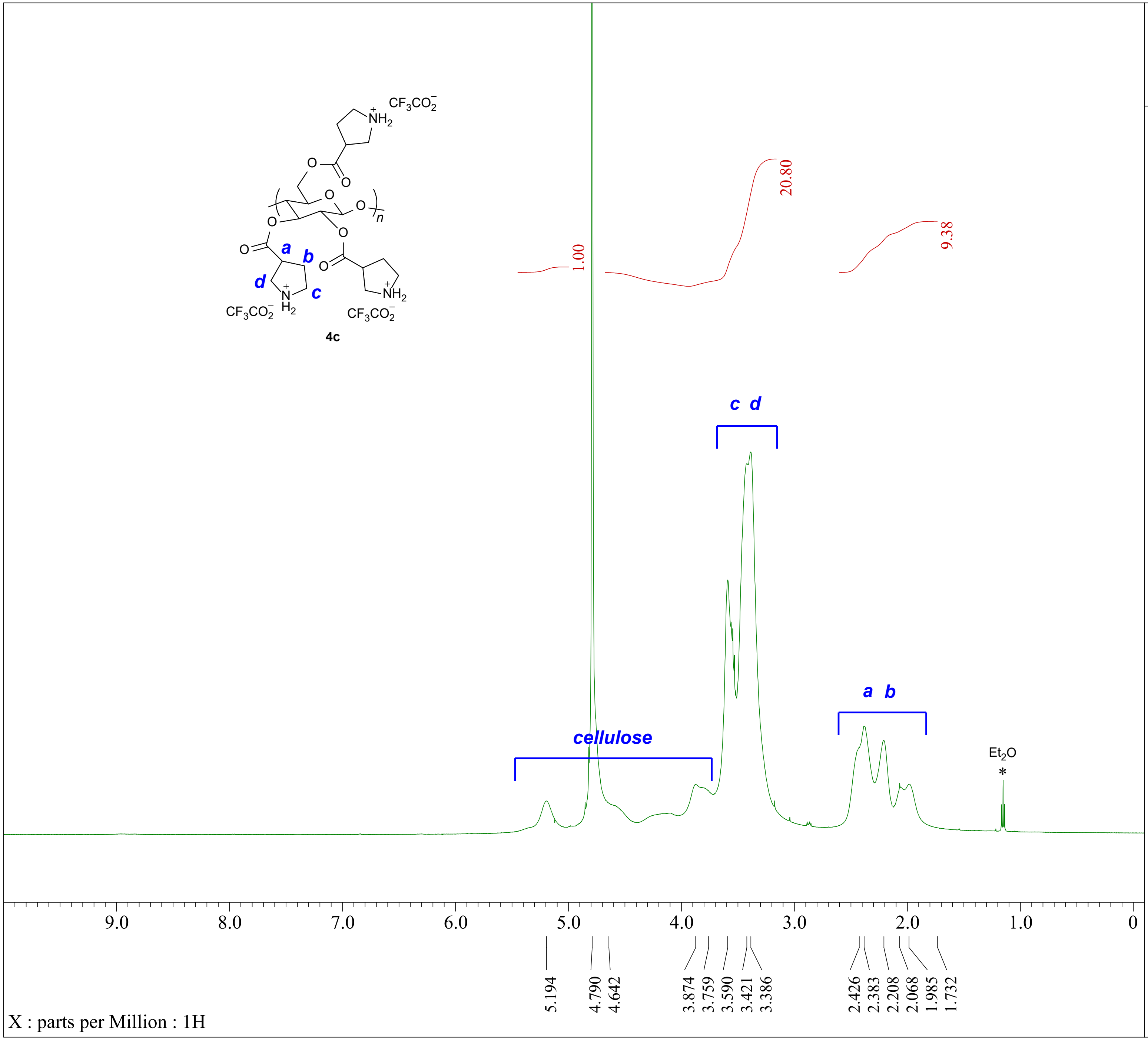


| | |
|---|---|
| Filename | = ME_1_64_Cy5-3TFA_ppt2-4.jd |
| Author | = delta |
| Experiment | = single_pulse.ex2 |
| Sample_Id | = S#326593 |
| Solvent | = D2O |
| Creation_Time | = 22-APR-2020 08:50:28 |
| Revision_Time | = 4-JUN-2020 14:07:47 |
| Current_Time | = 4-JUN-2020 14:08:15 |
| Data_Format | = 1D COMPLEX |
| Dim_Size | = 13107 |
| Dim_Title | = 1H |
| Dim_Units | = [ppm] |
| Dimensions | = X |
| Site | = ECA 500 |
| Spectrometer | = JNM-ECA500 |
| Field_Strength | = 11.7473579[T] (500[MHz]) |
| X_Acq_Duration | = 1.74587904[s] |
| X_Domain | = 1H |
| X_Freq | = 500.15991521[MHz] |
| X_Offset | = 5.0[ppm] |
| X_Points | = 16384 |
| X_Prescans | = 1 |
| X_Resolution | = 0.57277737[Hz] |
| X_Sweep | = 9.38438438[kHz] |
| Irr_Domain | = 1H |
| Irr_Freq | = 500.15991521[MHz] |
| Irr_Offset | = 5.0[ppm] |
| Tri_Domain | = 1H |
| Tri_Freq | = 500.15991521[MHz] |
| Tri_Offset | = 5.0[ppm] |
| Clipped | = FALSE |
| Scans | = 64 |
| Total_Scans | = 64 |
| Relaxation_Delay | = 2[s] |
| Recvr_Gain | = 36 |
| Temp_Get | = 20.6[dC] |
| X_90_Width | = 11.4[us] |
| X_Acq_Time | = 1.74587904[s] |
| X_Angle | = 45[deg] |
| X_Atn | = 3.6[dB] |
| X_Pulse | = 5.7[us] |
| Irr_Mode | = Off |
| Tri_Mode | = Off |
| Dante_Presat | = FALSE |
| Initial_Wait | = 1[s] |
| Repetition_Time | = 3.74587904[s] |

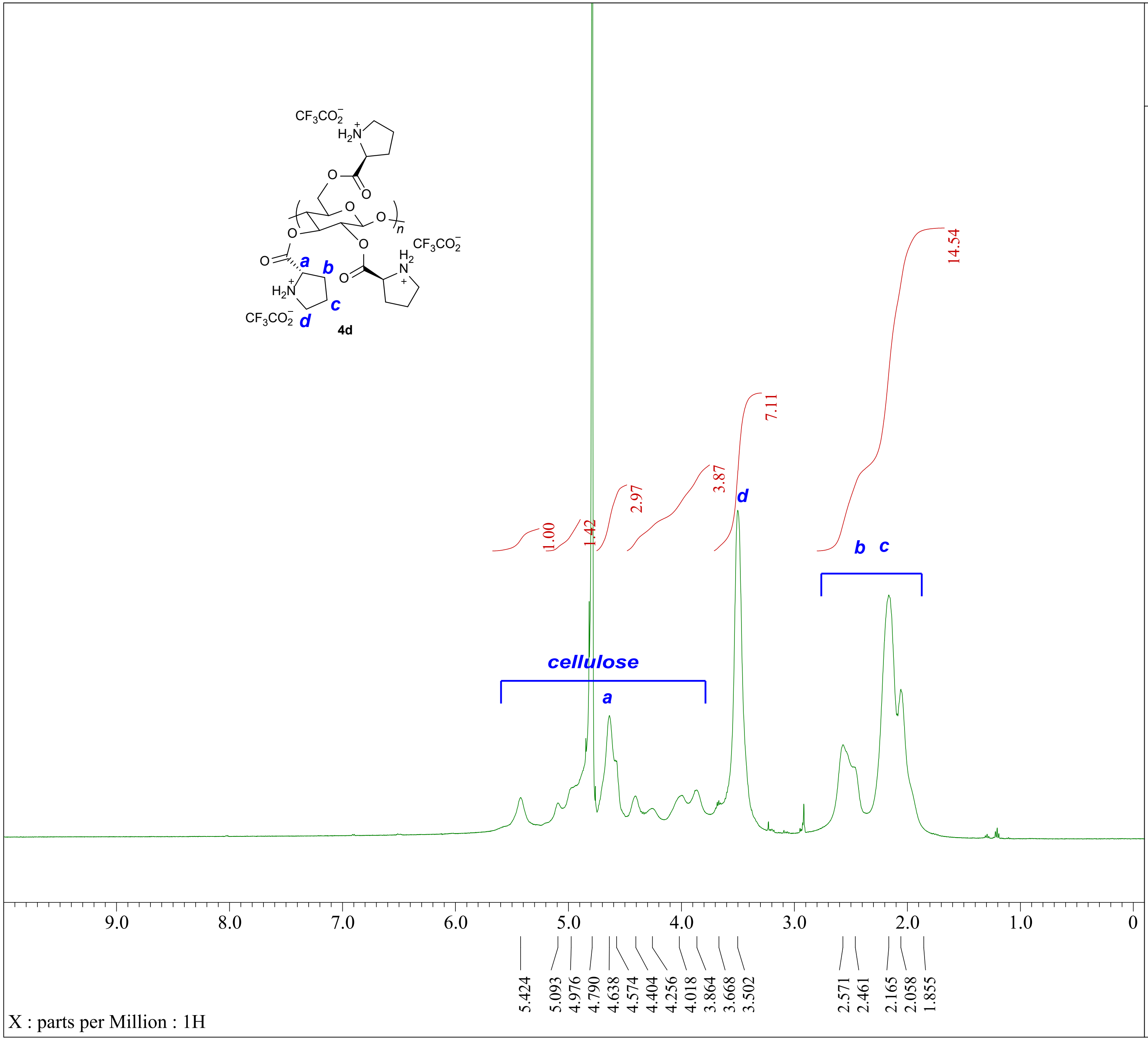


```
Filename           = ME1_66_Pro_TFA_ppt2-4.jdf
Author             = delta
Experiment         = single_pulse.ex2
Sample_Id          = S#340008
Solvent            = D2O
Creation_Time      = 27-APR-2020 09:12:52
Revision_Time      =  4-JUN-2020 14:39:03
Current_Time       =  4-JUN-2020 14:39:39

Data_Format        = 1D COMPLEX
Dim_Size           = 13107
Dim_Title          = 1H
Dim_Units          = [ppm]
Dimensions         = X
Site               = ECA 500
Spectrometer       = JNM-ECA500

Field_Strength     = 11.7473579[T] (500[MHz])
X_Acq_Duration     = 1.74587904[s]
X_Domain           = 1H
X_Freq             = 500.15991521[MHz]
X_Offset           = 5.0[ppm]
X_Points           = 16384
X_Prescans         = 1
X_Resolution       = 0.57277737[Hz]
X_Sweep            = 9.38438438[kHz]
Irr_Domain         = 1H
Irr_Freq           = 500.15991521[MHz]
Irr_Offset         = 5.0[ppm]
Tri_Domain         = 1H
Tri_Freq           = 500.15991521[MHz]
Tri_Offset         = 5.0[ppm]
Clipped            = FALSE
Scans              = 64
Total_Scans        = 64

Relaxation_Delay   = 2[s]
Recvr_Gain         = 40
Temp_Get           = 25.2[dC]
X_90_Width         = 11.4[us]
X_Acq_Time         = 1.74587904[s]
X_Angle            = 45[deg]
X_Atn              = 3.6[dB]
X_Pulse            = 5.7[us]
Irr_Mode           = Off
Tri_Mode           = Off
Dante_Presat       = FALSE
Initial_Wait       = 1[s]
Repetition_Time    = 3.74587904[s]
```

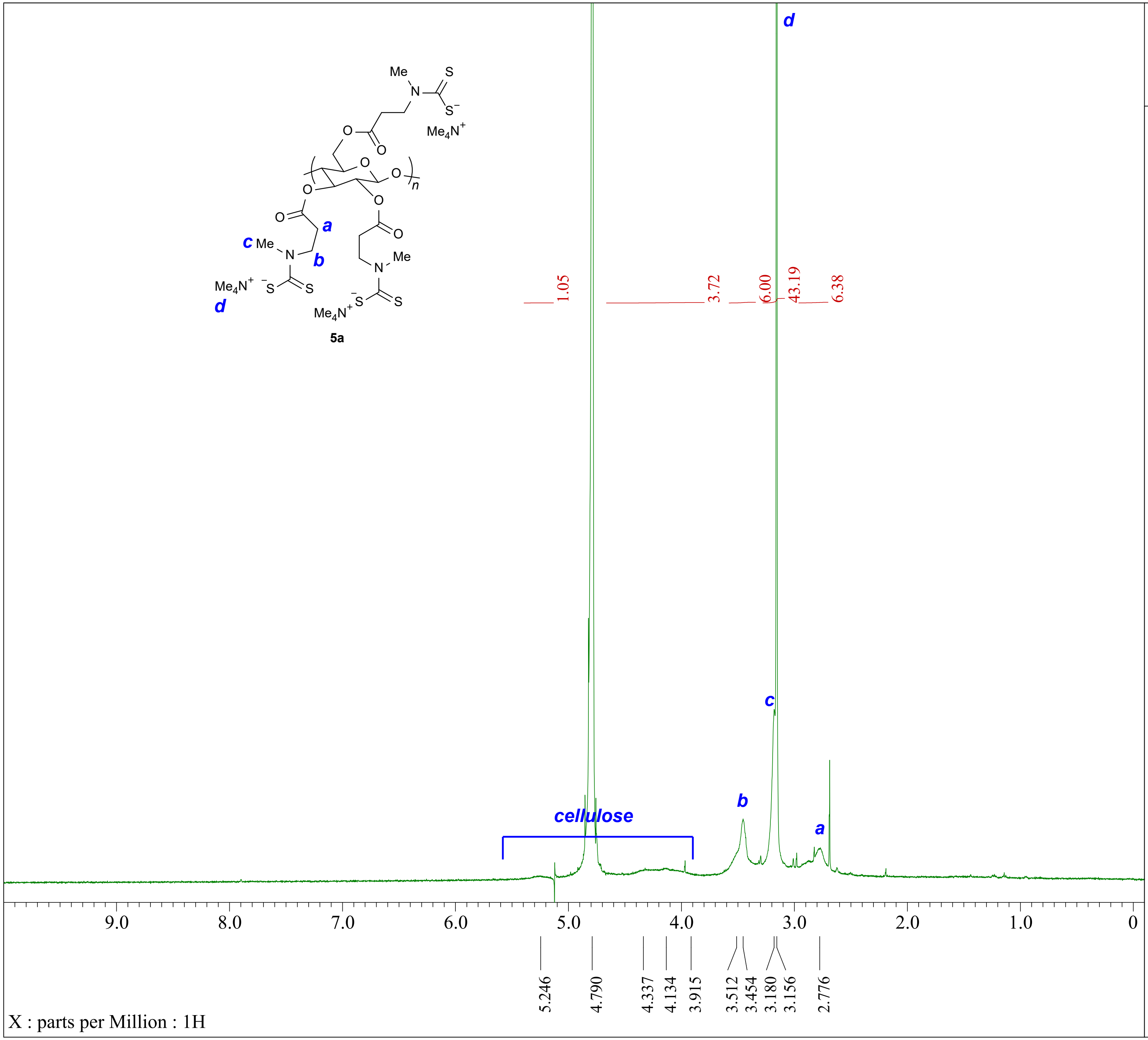


JEOL
Solutions for Innovation

```
Filename          = 5a-2.jdf
Author            = delta
Experiment        = single_pulse.ex2
Sample_Id         = S#683558
Solvent           = D2O
Creation_Time     =  7-MAR-2020 18:58:57
Revision_Time     =  5-JUN-2020 15:45:34
Current_Time      =  5-JUN-2020 15:46:13

Comment           = FM115-cel-2mnm-dtc-dry
Data_Format       = 1D COMPLEX
Dim_Size          = 13107
Dim_Title         = 1H
Dim_Units         = [ppm]
Dimensions        = X
Site              = ECA 500
Spectrometer      = JNM-ECA500

Field_Strength    = 11.7473579[T] (500[MHz])
X_Acq_Duration    = 1.74587904[s]
X_Domain          = 1H
X_Freq            = 500.15991521[MHz]
X_Offset          = 5.0[ppm]
X_Points          = 16384
X_Prescans        = 1
X_Resolution      = 0.57277737[Hz]
X_Sweep           = 9.38438438[kHz]
Irr_Domain        = 1H
Irr_Freq          = 500.15991521[MHz]
Irr_Offset        = 5.0[ppm]
Tri_Domain        = 1H
Tri_Freq          = 500.15991521[MHz]
Tri_Offset        = 5.0[ppm]
Clipped           = FALSE
Scans             = 256
Total_Scans       = 256

Relaxation_Delay  = 2[s]
Recvr_Gain        = 54
Temp_Get          = 20.1[dC]
X_90_Width        = 11.6[us]
X_Acq_Time        = 1.74587904[s]
X_Angle           = 45[deg]
X_Atn             = 3.6[dB]
X_Pulse           = 5.8[us]
Irr_Mode          = Off
Tri_Mode          = Off
Dante_Presat      = FALSE
Initial_Wait      = 1[s]
Repetition_Time   = 3.74587904[s]
```

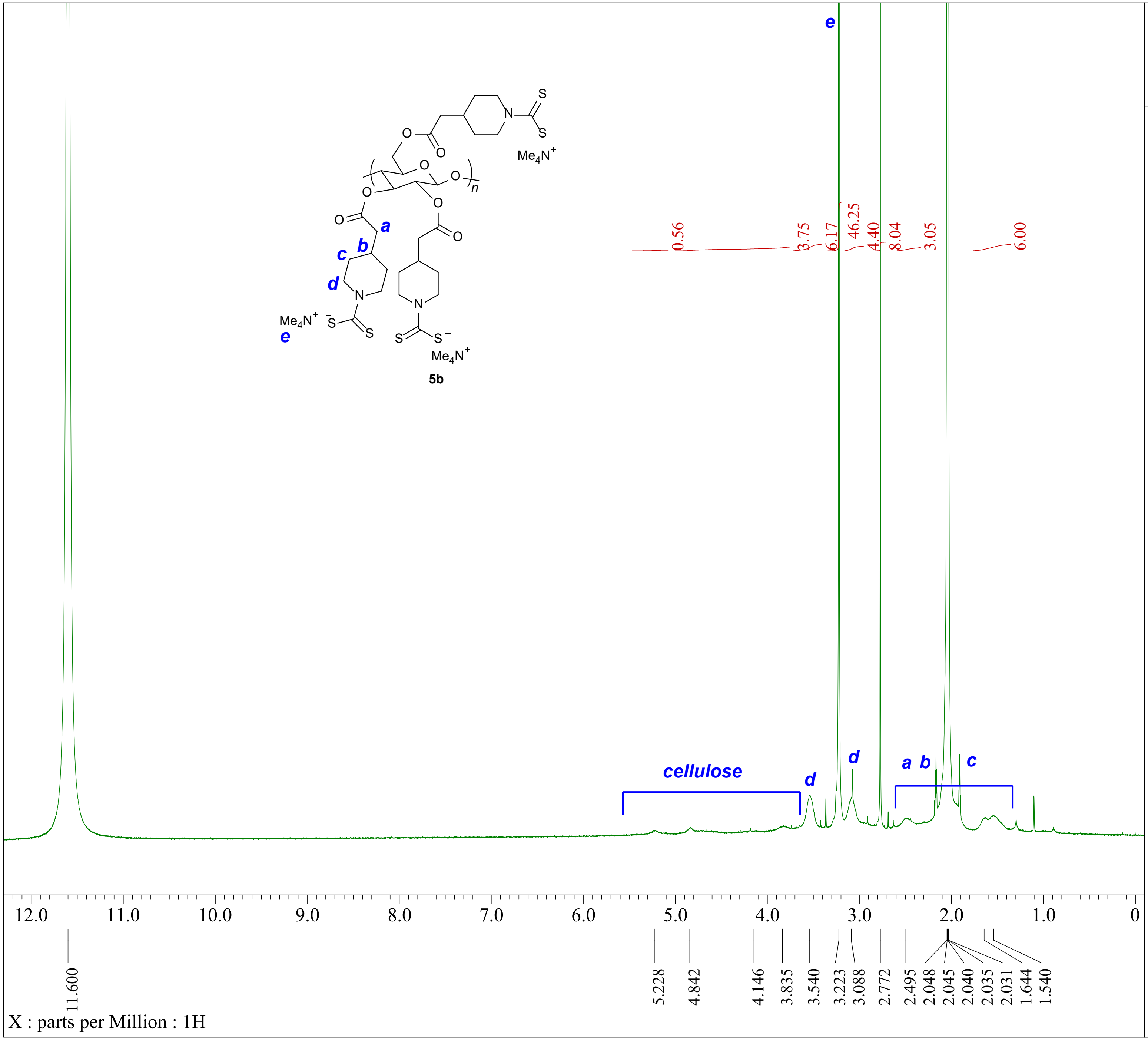


| | |
|---|---|
| Filename | = FM30-cel-pa-dtc-dry-7.jdf |
| Author | = delta |
| Experiment | = single_pulse.ex2 |
| Sample_Id | = S#714873 |
| Solvent | = ACETIC_ACID-D3 |
| Creation_Time | = 18-JUL-2019 20:03:18 |
| Revision_Time | = 4-JUN-2020 16:05:15 |
| Current_Time | = 4-JUN-2020 16:08:45 |
| Data_Format | = 1D COMPLEX |
| Dim_Size | = 13107 |
| Dim_Title | = 1H |
| Dim_Units | = [ppm] |
| Dimensions | = X |
| Site | = ECA 500 |
| Spectrometer | = JNM-ECA500 |
| Field_Strength | = 11.7473579[T] (500[MHz]) |
| X_Acq_Duration | = 1.74587904[s] |
| X_Domain | = 1H |
| X_Freq | = 500.15991521[MHz] |
| X_Offset | = 5.0[ppm] |
| X_Points | = 16384 |
| X_Prescans | = 1 |
| X_Resolution | = 0.57277737[Hz] |
| X_Sweep | = 9.38438438[kHz] |
| Irr_Domain | = 1H |
| Irr_Freq | = 500.15991521[MHz] |
| Irr_Offset | = 5.0[ppm] |
| Tri_Domain | = 1H |
| Tri_Freq | = 500.15991521[MHz] |
| Tri_Offset | = 5.0[ppm] |
| Clipped | = FALSE |
| Scans | = 256 |
| Total_Scans | = 256 |
| Relaxation_Delay | = 2[s] |
| Recvr_Gain | = 50 |
| Temp_Get | = 21[dC] |
| X_90_Width | = 11.6[us] |
| X_Acq_Time | = 1.74587904[s] |
| X_Angle | = 45[deg] |
| X_Atn | = 3.6[dB] |
| X_Pulse | = 5.8[us] |
| Irr_Mode | = Off |
| Tri_Mode | = Off |
| Dante_Presat | = FALSE |
| Initial_Wait | = 1[s] |
| Repetition_Time | = 3.74587904[s] |

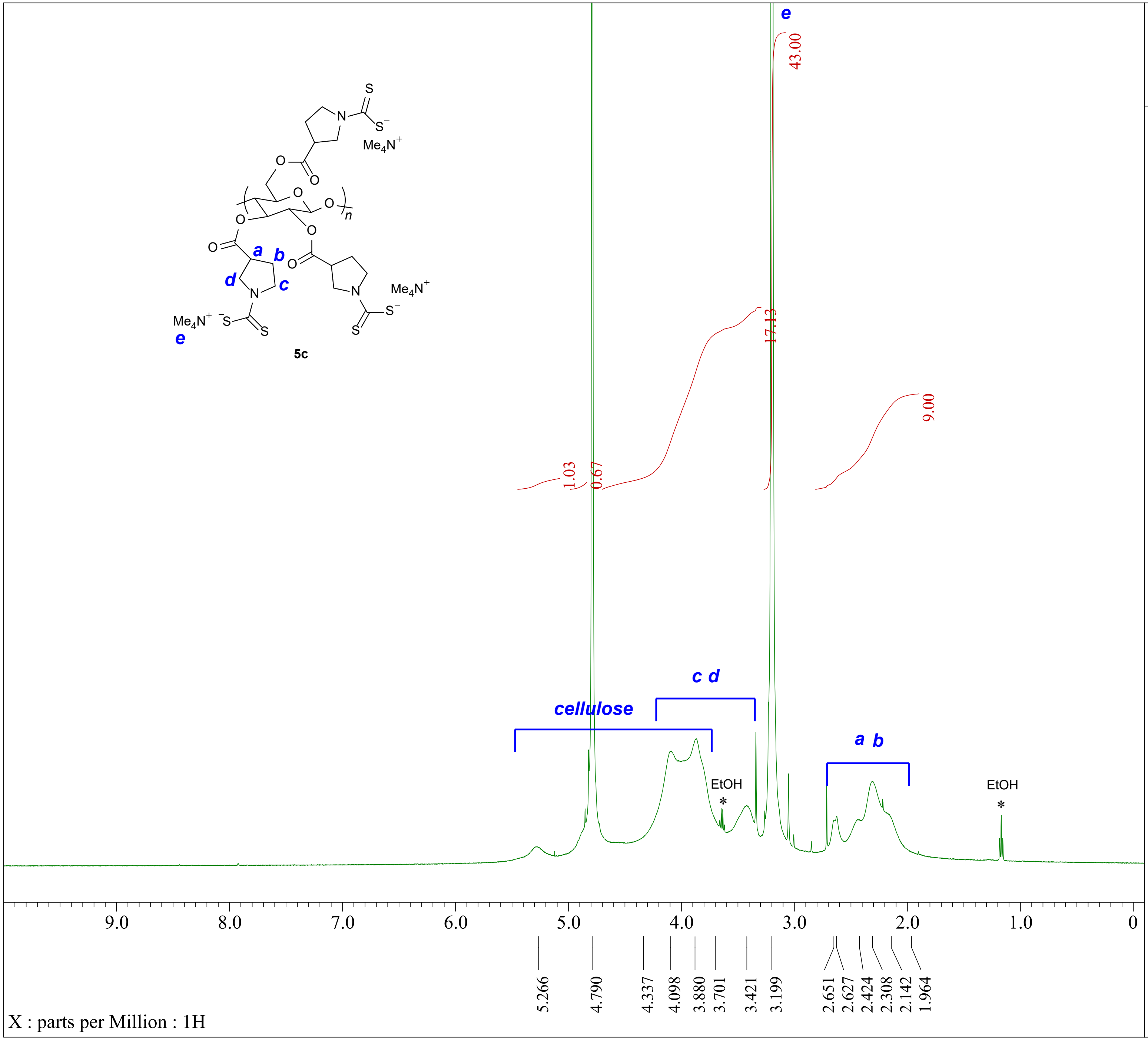


| | |
|---|---|
| Filename | = FM36-cel-prc-dtc-r.t.-4.jd |
| Author | = delta |
| Experiment | = single_pulse.ex2 |
| Sample_Id | = S#499551 |
| Solvent | = D2O |
| Creation_Time | = 13-SEP-2019 14:04:50 |
| Revision_Time | = 4-JUN-2020 22:44:01 |
| Current_Time | = 4-JUN-2020 22:44:06 |
| Comment | = FM36-cel-prc-dtc-r.t. |
| Data_Format | = 1D COMPLEX |
| Dim_Size | = 13107 |
| Dim_Title | = 1H |
| Dim_Units | = [ppm] |
| Dimensions | = X |
| Site | = ECA 500 |
| Spectrometer | = JNM-ECA500 |
| Field_Strength | = 11.7473579[T] (500[MHz]) |
| X_Acq_Duration | = 1.74587904[s] |
| X_Domain | = 1H |
| X_Freq | = 500.15991521[MHz] |
| X_Offset | = 5.0[ppm] |
| X_Points | = 16384 |
| X_Prescans | = 1 |
| X_Resolution | = 0.57277737[Hz] |
| X_Sweep | = 9.38438438[kHz] |
| Irr_Domain | = 1H |
| Irr_Freq | = 500.15991521[MHz] |
| Irr_Offset | = 5.0[ppm] |
| Tri_Domain | = 1H |
| Tri_Freq | = 500.15991521[MHz] |
| Tri_Offset | = 5.0[ppm] |
| Clipped | = FALSE |
| Scans | = 256 |
| Total_Scans | = 256 |
| Relaxation_Delay | = 2[s] |
| Recvr_Gain | = 46 |
| Temp_Get | = 21.6[dC] |
| X_90_Width | = 11.6[us] |
| X_Acq_Time | = 1.74587904[s] |
| X_Angle | = 45[deg] |
| X_Atn | = 3.6[dB] |
| X_Pulse | = 5.8[us] |
| Irr_Mode | = Off |
| Tri_Mode | = Off |
| Dante_Presat | = FALSE |
| Initial_Wait | = 1[s] |
| Repetition_Time | = 3.74587904[s] |

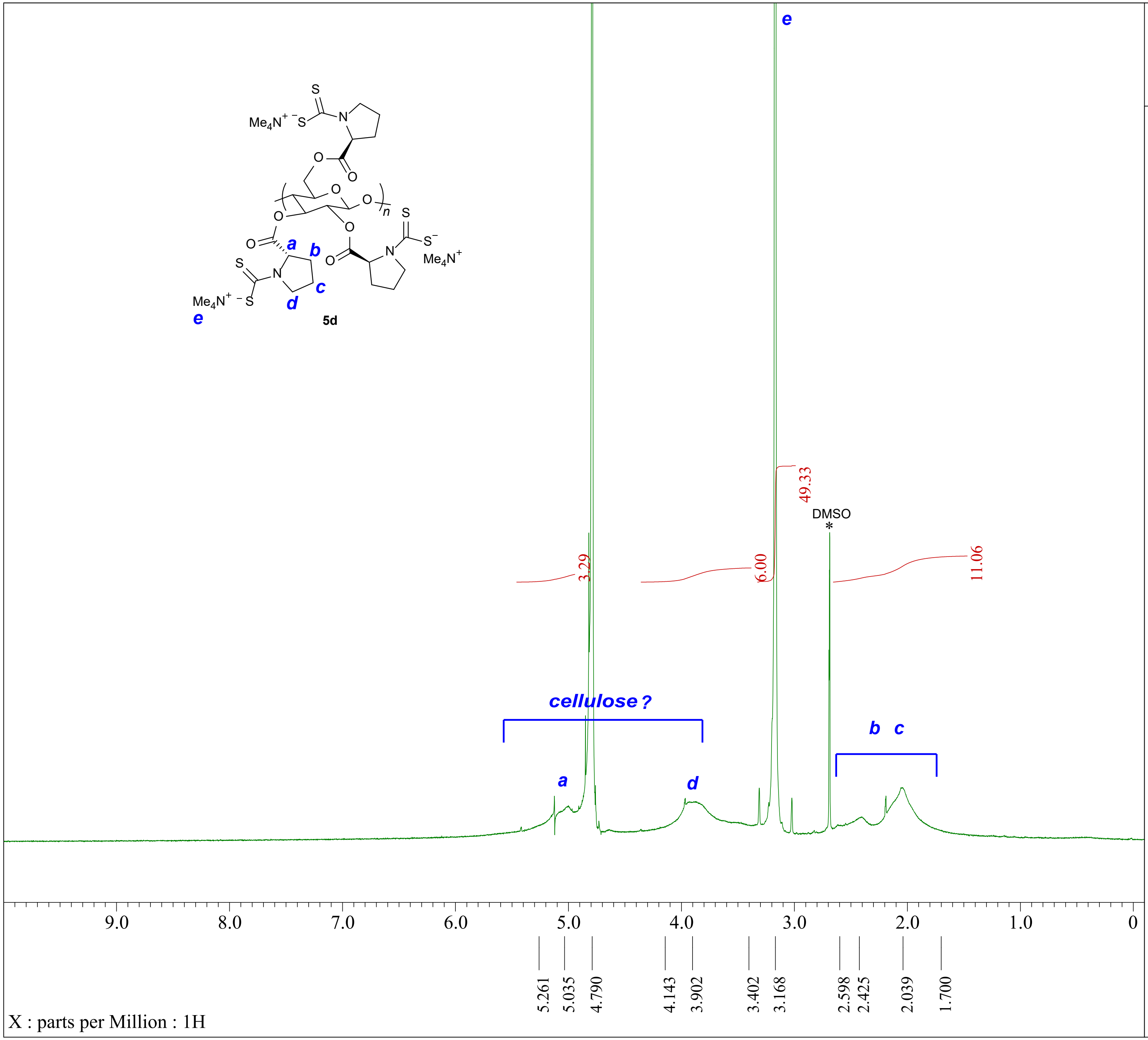


| | |
|---|---|
| Filename | = FM78-cel-pro-dtc-powder-dr |
| Author | = delta |
| Experiment | = single_pulse.ex2 |
| Sample_Id | = S#543947 |
| Solvent | = D2O |
| Creation_Time | = 13-FEB-2020 15:06:05 |
| Revision_Time | = 4-JUN-2020 22:53:48 |
| Current_Time | = 4-JUN-2020 22:54:14 |
| Data_Format | = 1D COMPLEX |
| Dim_Size | = 13107 |
| Dim_Title | = 1H |
| Dim_Units | = [ppm] |
| Dimensions | = X |
| Site | = ECA 500 |
| Spectrometer | = JNM-ECA500 |
| Field_Strength | = 11.7473579[T] (500[MHz]) |
| X_Acq_Duration | = 1.74587904[s] |
| X_Domain | = 1H |
| X_Freq | = 500.15991521[MHz] |
| X_Offset | = 5.0[ppm] |
| X_Points | = 16384 |
| X_Prescans | = 1 |
| X_Resolution | = 0.57277737[Hz] |
| X_Sweep | = 9.38438438[kHz] |
| Irr_Domain | = 1H |
| Irr_Freq | = 500.15991521[MHz] |
| Irr_Offset | = 5.0[ppm] |
| Tri_Domain | = 1H |
| Tri_Freq | = 500.15991521[MHz] |
| Tri_Offset | = 5.0[ppm] |
| Clipped | = FALSE |
| Scans | = 256 |
| Total_Scans | = 256 |
| Relaxation_Delay | = 2[s] |
| Recvr_Gain | = 50 |
| Temp_Get | = 20[dC] |
| X_90_Width | = 11.6[us] |
| X_Acq_Time | = 1.74587904[s] |
| X_Angle | = 45[deg] |
| X_Atn | = 3.6[dB] |
| X_Pulse | = 5.8[us] |
| Irr_Mode | = Off |
| Tri_Mode | = Off |
| Dante_Presat | = FALSE |
| Initial_Wait | = 1[s] |
| Repetition_Time | = 3.74587904[s] |
